# Low-dose ganciclovir ameliorates dextran sulfate sodium-induced ulcerative colitis through inhibiting macrophage STING activation in mice

**DOI:** 10.3389/fphar.2022.1020670

**Published:** 2022-11-17

**Authors:** Lin-Kong Gong, Xiaodong Yang, Juan Yang, Shu Wu, Yue Chen, Jiang-Tao Zhang, Zhi-Hong Wang, Li-Hua Chen, Chungen Xing, Tong Liu

**Affiliations:** ^1^ Department of General Surgery, The Second Affiliated Hospital of Soochow University, Suzhou, China; ^2^ Institute of Pain Medicine and Special Environmental Medicine, Nantong University, Nantong, China; ^3^ Jiangsu Key Laboratory of Neuropsychiatric Diseases and Institute of Neuroscience, Soochow University, Suzhou, China; ^4^ Department of Nutrition and Food Hygiene, School of Public Health, Nantong University, Nantong, China; ^5^ College of Life Sciences, Yanan University, Yanan, China; ^6^ Suzhou Key Laboratory of Intelligent Medicine and Equipment, Suzhou, China

**Keywords:** ganciclovir, colitis, microbiome, STING, macrophage

## Abstract

Ganciclovir (GCV) is a prodrug nucleoside analogue and is clinically used as antiviral drug for the treatment of cytomegalovirus (CMV) and other infections. Based on the potential anti-inflammatory activity of GCV, this study aimed to investigate the therapeutic effects of ganciclovir on dextran sulfate sodium (DSS)-induced ulcerative colitis (UC), which may involve cyclic GMP-AMP synthase (cGAS)-stimulator of interferon genes (STING) pathways. Our results demonstrated that incubation of GCV (50 μM) inhibited cGAS-STING pathway in macrophage RAW264.7 cells. Then, it was found that intestinal cGAS-STING pathways were upregulated in UC patients, Crohn’s disease colitis (CD) patients, and DSS-induced colitis mice. Intraperitoneal injection of low-dose GCV (10 mg/kg/day) attenuated DSS-induced colitis and abdominal pain in mice. GCV treatment significantly inhibited the upregulation of cGAS-STING pathway in DSS-induced colitis mice. Moreover, DSS-induced colitis and gut dysbiosis was markedly attenuated in STING deficient mice compared with that of wild-type (WT) mice. Finally, there was lacking therapeutic effect of GCV on DSS-induced colitis in STING deficient mice. Together, our results indicated that low-dose GCV ameliorated DSS-induced UC in mice, possibly through inhibiting STING signaling in colonic macrophages, indicating that GCV may be useful for the treatment of UC.

## Introduction

Ganciclovir (GCV) is a widely used antiviral drug and a close analog of acyclovir, the first successful antiviral drug, described in 1977 to exploit viral thymidine kinase activity and inhibit viral replication (Elion et al., 1977). Today, GCV remains the drug of choice for the prevention and treatment of cytomegalovirus (CMV) infection in transplant recipients (Scott et al., 2004). The canonical function of GCV to inhibit virus replication requires intracellular phosphorylation by viral thymidine kinase (tk) to the triphosphate derivative, which is a competitive inhibitor of deoxyguanosine triphosphate thereby impairing viral DNA synthesis (Skripuletz et al., 2015). After decades of high-efficiency use in humans, besides its potent effects on viral replication, it has recently shown that GCV has a significant atypical anti-inflammatory activity. GCV at therapeutic concentrations also inhibits proliferation of uninfected cells, most notably of bone marrow cells, possibly by targeting endogenous tk or through other unknown mechanisms (Ding et al., 2014). Notably, it was recently demonstrated that the type I interferon response in microglia induced by GCV was not mediated through stimulator of interferon genes (STING), attributing to the ability of GCV to reduce neuroinflammation in the cultured microglia and in a mouse model of multiple sclerosis (experimental autoimmune encephalomyelitis, EAE) (Mathur et al., 2017). It was also demonstrated that GCV reduced irinotecan-induced intestinal toxicity by inhibiting NOD-, LRR- and pyrin domain-containing protein 3 (NLRP3) inflammasomes (Huang et al., 2020). Together, it was proposed that repurposing anti-viral drug GCV may be a promising strategy for anti-inflammatory therapy.

Inflammatory bowel disease (IBD) is a chronic relapsing and remitting inflammatory diseases of the gastrointestinal tract, which is associated with dysfunction of gut immune system and the commensal ecosystems (Graham and Xavier, 2020). The incidences of IBD are increasing worldwide. However, the pathogenesis of IBD is incompletely understood, which involves complex interactions of genetic, environmental, and immunoregulatory factors (Jostins et al., 2012). IBD is classified into ulcerative colitis (UC) and Crohn’s disease (CD). UC mainly affects the colon, while CD may affect any region of the gastrointestinal tract. The main clinical manifestations of IBD are abdominal pain, diarrhea, rectal bleeding, weight loss, fever, and fatigue (Ordás et al., 2012). Clinically, the present treatment of IBD are 5-salicylic acid inhibitors, corticosteroids and immunosuppressants. Moreover, some new targeted drugs such as infliximab (a monoclonal antibody against tumor necrosis factor-α /TNF-α), ustekinumab (a monoclonal antibody against the p40 subunit of interleukin 12/23), and adalimumab (a fully human neutralizing anti-TNFα monoclonal antibody) are also used to treat IBD patients (Fiske et al., 2022; Lerang et al., 2022; Song et al., 2022; Wasserbauer et al., 2022; Yao et al., 2022). Animal models of colitis have been established to investigate its mechanisms and are used to evaluate efficacy of potential anti-inflammatory agents. Especially, acute dextran sulfate sodium (DSS)-induced UC mouse model was widely used to study the contribution of innate immune mechanisms to intestinal inflammation, due to the toxicity of DSS on mucosa (Xavier and Podolsky, 2007).

STING (also known as TMEM173) is an adaptor protein located on the endoplasmic reticulum (ER) membrane and is involved in inflammatory responses. Cyclic GMP-AMP synthetase (cGAS) acts as a DNA sensor, including bacterial DNA, viral DNA, genomic DNA, and mitochondrial DNA. Upon DNA binding to cGAS, cGAS catalyzes ATP and GTP to cGAMP, which activates the ER adaptor protein STING. STING then recruits and activates TANK-binding kinase 1 (TBK1), and further phosphorylate interferon-regulated transcription factor 3 (IRF3) to induce the production of type I interferon and proinflammatory cytokines (Bai and Liu, 2019). In contrast with its role in host defense (Burdette and Vance, 2013; Schoggins et al., 2014; Zhu et al., 2014), excessive cGAS-STING activation also contributes to diverse pathological conditions. For example, it was observed that sepsis severity is reduced in STING-deficient mice relative to wild-type (WT) mice in the cecal ligation and puncture model of sepsis (Heipertz et al., 2017; Zeng et al., 2017; Hu et al., 2019). Acute or chronic carbon tetrachloride-induced hepatocyte death and fibrosis were prevented in STING deficient mice compared with that of WT mice (Petrasek et al., 2013). In a murine model of cytosolic self-DNA-mediated autoimmunity, STING deficiency also reduces pro-inflammatory cytokines and arthritis scores (Ahn et al., 2012). In addition, STING gain-of-function mutations lead to an auto-immune disease (Liu et al., 2014). Thus, inhibition of excessive activation of STING may be beneficial for many inflammatory diseases.

In the present study, we tested a hypothesis that GCV may have therapeutic effects on DSS-induced colitis in mice, possible through acting on cGAS-STING signaling.

## Materials and methods

### IBD data analysis

A meta-analysis of 6 published IBD studies based on microarrays was conducted (Supplementary Table S1). That dataset with a total of 364 IBD tissue samples (UC or CD) and 86 controls. We compared diseases based on categorizations defined in each study in the meta-analysis. In general, our selection criteria include 1) Samples from inflamed tissues are used for disease analysis when disease samples include inflamed tissues and non-inflamed tissues 2) Samples prior to treatment as the disease group was used if any treatment was included in disease samples (3 studies: GSE16879; GSE73661; GSE52746); and 3) Samples from active patients were used as disease groups if the samples included both active and inactive patients (4 studies: GSE59071; GSE75214; GSE52746; GSE37283). We used the R Affy package to process microarray studies and applied quantile normalization to adjust between the array baseline biases. A STAR alignment (v 2.5.2b) was performed to map NGS datasets to GRCh37.75, the reference genome for humans. The gene counts matrix was generated later using the featureCounts tool from the Subread package (v 1.6.0). For microarray datasets, GLM model from R’s limma package (v. 3.1) was used to calculate differentially expressed genes between disease patients (UC or CD) and healthy controls. The analysis was conducted with genes with fold changes of 1.5 and adjusted p-values of 0.05 (Cheng et al., 2022).

### Animals

Male C57BL/6J mice (6–8 weeks old) were obtained from the Shanghai SLAC Laboratory Animal CO., LTD. (Shanghai, China). We randomly assigned male C57BL/6J mice of matched age to the different group. The group of mice during testing was blinded to the experimenter of the behavioral test. Male STING deficient mice (STING ^gt/gt^) were purchased from the Jackson Laboratories (Bar Harbor, ME, USA), and raised at Soochow University Laboratory Animal Center. All animals were kept on a 12 h light/12 h night cycle. The rearing environment was maintained at a constant room temperature of 22 °C and 60%–80% humidity. In addition, water and food were freely available. This animal study followed the ARRIVE guidelines (Kilkenny et al., 2010).

### Human subjects

The study was approved by the Institutional Ethics Committee of the Second Affiliated Hospital of Soochow University. Total 4 UC patients and 4 colon cancer patients were used. The marginal non-cancer colon tissues were used as the non-UC controls (the Normal group). Biopsies were collected to use for Immunohistochemistry. The demographic data are presented in Supplementary Table S2. Written informed consent was obtained from all participants.

#### Classification according to disease extent

Colonoscopy can be used to classify patients with UC according to the macroscopic extent of disease. 40% of patients with UC have disease limited to the rectum (ulcerative proctitis), 30%–40% to the rectosigmoid colon (ulcerative proctosigmoiditis) or the left-colon (left-sided UC), and 20%–30% have disease extending proximal to the splenic flexure or involving the entire colon (extensive UC or substantial UC and pancolonic or universal UC). Patients with ulcerative proctitis and ulcerative proctosigmoiditis are collectively termed distal UC (Ekbom et al., 1991; Langholz et al., 1991; D'Haens et al., 2007).

Patients with UC have a spectrum of disease severity ranging from remission to severely active. Clinical assessment can be used to classify UC patients into 4 disease activity subgroups: 1) remission (≤2 or 3 stools/day, without the presence of blood and/or pus in the stools, with no systemic symptoms); 2) mildly active disease (3 or 4 stools/day and/or presence of blood and/or pus in the stools less than daily, with no systemic symptoms of fever or weight loss); 3) moderately active disease (>4 stools/day and/or daily presence of blood and/or pus) with minimal systemic symptoms; and 4) severely active disease (>6 bloody stools/day, and evidence of toxicity, as demonstrated by fever, tachycardia, anemia, or an ESR) (note that the stool frequency for remission and mild disease may overlap the upper limit of normal stool frequency). (Langholz et al., 1994; Kornbluth and Sachar, 2004; Liu et al., 2021).

### Dextran sulfate sodium-induced colitis mouse models

Male mice (ages 6–8 weeks) used for these experiments were maintained under specific pathogen-free conditions and were free from *Helicobacter*, *Citrobacter* *rodentium*, and *Norovirus*. Acute experimental colitis was induced by the addition of 3% (w/v) Dextran Sulfate Sodium Salt (MP 160110; molecular weight [MW], 36,000-5,000) *ad libitum* into the normal drinking water of mice. Mice were provided normal pelleted diet *ad libitum* during experimentation. Mice were treated for 8 days with 3% DSS with replacement of the 3% DSS solution every 2 days. As previously described, during each day of the experimental course clinical parameters for disease severity were assessed. Mice were weighed for body weight loss evaluation and feces was assessed for fecal blood and diarrhea (Ahn et al., 2017).

Chronic colitis was induced by addition of 3% (w/v) colitis grade dextran sulfate sodium (DSS) with molecular weight 36,000-50,000, to the drinking water for 7 days followed by a 7-day recovery period. This treatment schedule was repeated for 3 cycles and is suited to induce a consistent and reproducible state of chronic colitis in mice. Body weight was monitored daily. Mice developed moderate colitis, manifested with typical clinical symptoms such as weight loss, diarrhea, and rectal bleeding. All animal experiments were performed according to the randomized block experimental design (Vlantis et al., 2016; Wirtz et al., 2017; Wang Y. et al., 2018). The detailed experimental procedures were shown in Supplementary Figure S1.

### Measurement of referred visceral mechanical sensitivity

Using von Frey filaments the visceral hypersensitivity of the abdominal region was evaluated on Day 8. Abdominal allodynia was assessed using the “up-and-down” methods. Each mice were placed beneath perspex boxes (10 × 10 × 7 cm) set upon elevated wire mesh stands and acclimated for 30 min. Von Frey filaments were applied to the abdomen (between diaphragm and genitals). Von Frey filament (0.008–0.6 g) was applied to the abdomen area with enough pressure to bend the filament. The filament was held for 3 s. If the abdomen did not lift after 3 s, an increased weight filament would be used next. Whereas a subsequently weaker filament would be used if the abdomen lifted after filament stimuli. The maximal duration of each force application was 3 s, and the inter-stimulus interval was 2–3 min. Following each challenge, the withdrawal response was quantified either as 1 (withdrawal or licking) or 0 (no response). The 50% mechanical abdomen withdrawal threshold was collected. The abdomen mechanical withdraw thresholds were recorded in grams, and they were detected before (baseline) after DSS treatment, and after GCV treatments (1, 3, 5, and 7 Day). Chronic colitis mice were tested once a week (Wang C. Z. et al., 2018).

### Evaluation of disease activity index

The mice were checked daily for colitis based on body weight, gross rectal bleeding, and stool consistency. A disease activity index (DAI) score was calculated according to a described method to assess the disease severity (Chaudhary et al., 2017).

### Western blotting analysis

RAW264.7 cells (CLS Cat#400319/p462_RAW-2647, RRID: CVCL_0493; Passage number <20) were plated at a density of 1.5 × 10^5^ cells per 6 cm dish. After at least 12 h, the cells were incubated with GCV (50 μM) for 24 h at 37°C. Then, cells were lysed with Radio-Immunoprecipitation Assay (RIPA) (RIPA) buffer containing a cocktail of phosphatase inhibitors and protease inhibitors after washed with Phosphate Buffered Saline (PBS). On day 8 after GCV injection, the mice were under deep anesthesia with isoflurane, intracardiac perfusion was performed with 0.9% saline. The Colon tissue were also rapidly collected and homogenized in lysis buffer containing a cocktail of phosphatase inhibitors and protease inhibitors for total protein extraction assays. The protein concentrations were measured by Pierce bicinchoninic acid (BCA) protein assay (ThermoFisher Scientific, Cat#23250; Waltham, MA, USA), and equal amounts of protein (25 μg) were loaded onto each lane and separated on 10% sodium dodecyl-sulfate polyacrylamide gel electrophoresis (SDS-PAGE). After transfer, the blots were blocked with 5% nonfat milk diluted in Tris-HCl Buffer Saline (TBS) at room temperature for 1 h and the PVDF membranes were incubated overnight at 4°C with primary monoclonal anti-STING (Novus Cat#NBP2-24683, RRID: AB_2868483), cGAS (Cell Signaling Technology Cat#15102, RRID: AB_2732795), p-TBK1 (Cell Signaling Technology Cat# 5483, RRID: AB_10693472), TBK1 (Abcam Cat#ab40676, RRID: AB_776632), IFN-β (Abcam, Cat#ab65783, RRID: AB_1658870), TNF-α (Abcam, Cat#ab205587, RRID: AB_2889389), IL-1β (Cell Signaling Technology, Cat#12242, RRID: AB_2715503). For loading control, the blots were probed with β-Actin antibody (ImmunoWay Cat#YT0099, RRID: AB_2885029) and β-Tubulin antibody (Affinity Biosciences Cat#T0023, RRID: AB_2813772). The blots were washed and incubated with horseradish peroxidase-conjugated goat anti-mouse IgG secondary antibody (Thermo Fisher Scientific Cat# G-21040, RRID: AB_2536527) and goat anti-rabbit IgG secondary antibody (Thermo Fisher Scientific Cat#G-21234, RRID: AB_2536530) The washed protein bands were developed using ultrasensitive ECL chemiluminescence kit (NCMECL Ultra) and analyzed for grayscale values as indicated. The ratio was calculated and then normalized to the control measurements (Taylor and Posch, 2014). Data from four mice were used for statistical analysis.

### Quantitative real-time polymerase chain reaction

Total RNA was isolated from frozen tissues by guanidinium isothiocyanate-phenol extraction and quantified by measuring absorbance at 260 nm and 280 nm. 1 μg of total RNA was used for reverse transcription. STING, inflammatory factors mRNA were quantified by qPCR (prism 7,500; Applied Biosystems, Foster, California). Quantitative real-time polymerase chain reaction (qPCR) test was conducted by SYBR Green PCR Master Mix (Roche, Basel, Switzerland) using real-time PCR Detection System (ABI 7500, Life technology, USA). The cycling conditions included a 10-min initial denaturation step at 95°C followed by 40 cycles of 15 s at 95°C and 1 min at 60°C. Target gene expression was normalized to the housekeeper gene GAPDH expression. Relative fold difference in expression was calculated using 2^−ΔΔCT^ method after normalization to GAPDH expression. Triplicate RT-PCR analyses were performed (Green and Sambrook, 2018). Q-RT-PCR primer sequences (5′ to 3’) used were listed in Table 1:

**TABLE 1:** Q-RT-PCR primer sequences used in this study.

Primers	From 5′ to 3′
*Gapdh*-Mouse-Forward	GAA​GGT​CGG​TGT​GAA​CGG​AT
*Gapdh*-Mouse-Reverse	AAT​CTC​CAC​TTT​GCC​ACT​GC
*Cgas*-Mouse-Forward	GCC​GAG​ACG​GTG​AAT​AAA​GT
*Cgas*-Mouse-Reverse	CAT​TAG​GAG​CAG​AAA​TCT​TCA​CA
*Sting1*-Mouse-Forward	CGT​AGC​CTC​GCA​GCA​ACT​TG
*Sting1*-Mouse-Reverse	ACC​TGG​ACT​GGA​CAT​GGC​AC
*Il6*-Mouse-Forward	ACT​TCA​CAA​GTC​CGG​AGA​GG
*Il6*-Mouse-Reverse	TGC​AAG​TGC​ATC​ATC​GTT​GT
*Il1b*-Mouse-Forward	CTT​CAG​GCA​GGC​AGT​ATC​ACT​CAT
*Il1b*-Mouse-Reverse	TCT​AAT​GGG​AAC​GTC​ACA​CAC​CAG
*Tnf*-Mouse-Forward	CAT​GAG​CAC​AGA​AAG​CAT​GAT​CCG
*Tnf*-Mouse-Reverse	AAG​CAG​GAA​TGA​GAA​GAG​GCT​GAG
*Il10*-Mouse-Forward	GGA​CTT​TAA​GGG​TTA​CTT​GGG​TTG​CC
*Il10*-Mouse-Reverse	CAT​TTT​GAT​CAT​CAT​GTA​TGC​TTC​T
*Cxcl10*-Mouse-Forward	CCA​AGT​GCT​GCC​GTC​ATT​TTC
*Cxcl10*-Mouse-Reverse	GGC​TCG​CAG​GGA​TGA​TTT​CAA
*Ifnb1*-Mouse-Forward	CAG​CTC​CAA​GAA​AGG​ACG​AAC
*Ifnb1*-Mouse-Reverse	GGC​AGT​GTA​ACT​CTT​CTG​CAT

### Histology

On day 8 and 42 after 3% DSS treatment, Mice were anesthetized using isoflurane and perfused with physiological saline transcardially. Perfused with sterile saline throughout the body, followed by fixation with 4% paraformaldehyde. The colon of mice was also extracted. Later the colons were fixed with 4% paraformaldehyde in neutral buffer and embedded in paraffin. Then colon tissue was stained with hematoxylin and eosin (H&E) for histological examination. Immunohistochemistry sections were examined under a ZEISS fluorescence microscope (Carl Zeiss OPMI Pentero, Germany), images were taken and the sections were examined with NIH ImageJ software (NIH, Bethesda, MD) for analysis. For H&E staining analysis, several parameters were evaluated, including epithelium, well-defined crypt length, edema, neutrophil infiltration in mucosa and submucosa, and ulcers or erosions. And inflammation scores (0, normal, to 3, most severe) were calculated (Ahn et al., 2017).

### Immunofluorescence

On day 8 after GCV injection, the mice were completely anesthetized with isoflurane and then perfused with sterile saline throughout the body, followed by fixation with 4% paraformaldehyde. After fixation, the segment where the mouse colon was located was taken and the same fixative was left overnight. After sucrose gradient dehydration, the tissues were embedded with OCT tissue embedding agent and frozen at −80 °C in the refrigerator. The embedded tissues were sectioned (20 μm thickness) and processed on a cryostat microtome (CM 1950; Leica Microsystems, Wetzlar, Germany). Sections were incubated with 5% goat serum and incubated overnight at 4°C with primary anti F4/80 antibody (Abcam Cat#ab6640, RRID: AB_1140040) and anti-STING antibody (Novus Cat# NBP2-24683, RRID: AB_2868483). Then after washing away the first antibody, sections were incubated with FITC and Cy3 conjugated secondary antibodies (goat anti-rabbit Alex Flour 555 pAb Cell Signaling Technology Cat#4413, RRID: AB_10694110) (goat anti-rabbit Alex Flour 488 pAb Abcam Cat#ab150157, RRID: AB_2722511) for 1 h at room temperature (Ding et al., 2022). Immunostained sections were examined under a ZEISS fluorescence microscope (Carl Zeiss OPMI Pentero, Germany), images were taken and the sections were examined with NIH ImageJ software (NIH, Bethesda, MD) for analysis.

### Immunohistochemistry

The sections were incubated with 3% BSA (Cat#G5001, Servicebio), and incubated with the primary antibody STING (Novus Cat# NBP2-24683, RRID: AB_2868483) overnight at 4°C. Then the primary antibody was washed away, and the corresponding species of the secondary antibody was added dropwise. The genus secondary antibody (HRP labeled) covers the tissue. Washed off the secondary antibody (Millipore Cat# AP307P, RRID: AB_92641). After the slices were slightly dried, added freshly prepared DAB (Cat# G1211, Servicebio) color developing solution to the slice, controled the color development time under the microscope, and the result was brownish yellow. Rinsed the slices with tap water to wash color. Hematoxylin (Cat# G1004, Servicebio) was counter-stained for about 3 min, washed with tap water. Hematoxylin differentiation solution (Cat# G1039) was differentiated for a few seconds, and washed with tap water. Hematoxylin blue solution (G1040, Servicebio) returned to blue, and washed with running water. Microscopic examination (Carl Zeiss OPMI Pentero, Germany), image acquisition and analysis. The immunohistochemical results of paraffin section indicated that the hematoxylin-stained cell nucleus was blue, and the positive expression of DAB was brown-yellow. For IHC grading, the scores of positive staining in each field were defined as percentage of staining in the whole section, and the staining intensity is defined as no (0), weak (1), medium (2), and strong (3). The immunoscore was generated by multiplying these two scores (Miao et al., 2020).

### 16S rRNA sequencing analysis

We used the QIAamp DNA Mini Kit (QIAGEN, Germany) to extract total genomic DNA, following the manufacturer’s instructions. The bacterial 16S rRNA gene was amplified by PCR using the forward primer 343F (5′- TACGGRAGGCAGCAG -3′) and the reverse primer 798R (5′- AGGGTATCTAATCCT-3′) with the barcode and then sequenced using the Ion S5TMXL platform (Nossa et al., 2010).

### Reagents

Dextran Sulfate Sodium Salt (Cat# 0216011050), cGMP-AMP (Cat#531889), Lipopolysaccharide (Cat#L2880), DMXAA (Cat#D5817), CMA (Cat#S46701), ganciclovir (Cat#H20030419)was obtained from China keyi Pharmaceutical Co., Ltd. (Wuhan City, Hubei Province, China). Fecal occult blood test, F0BT (Cat#FD9349), other reagents were dissolved in sterile saline if not specified. All other chemicals used in the study were of analytical grade and obtained from commercial sources. The detailed regents used in this study were provided in Supplementary Table S3.

### Statistical analysis

The data was analyzed using Graph Prism 6 (Graph Pad, La Jolla, CA). Shapiro-Wilk test was used to test normality of the data. All values were presented as mean ± SEM. Unpaired Student’s *t*-test was used to compare two groups. Two-Way Repeated Measures ANOVA with post-hoc Bonferroni test was performed for multiple comparisons. Differences were considered statistically significant at *p* < 0.05. All statistical results were listed in Supplementary Table S4.

## Result

### Low-dose GCV inhibits cGAS-STING signaling in cultured RAW264.7 cells

**FIGURE 1:**
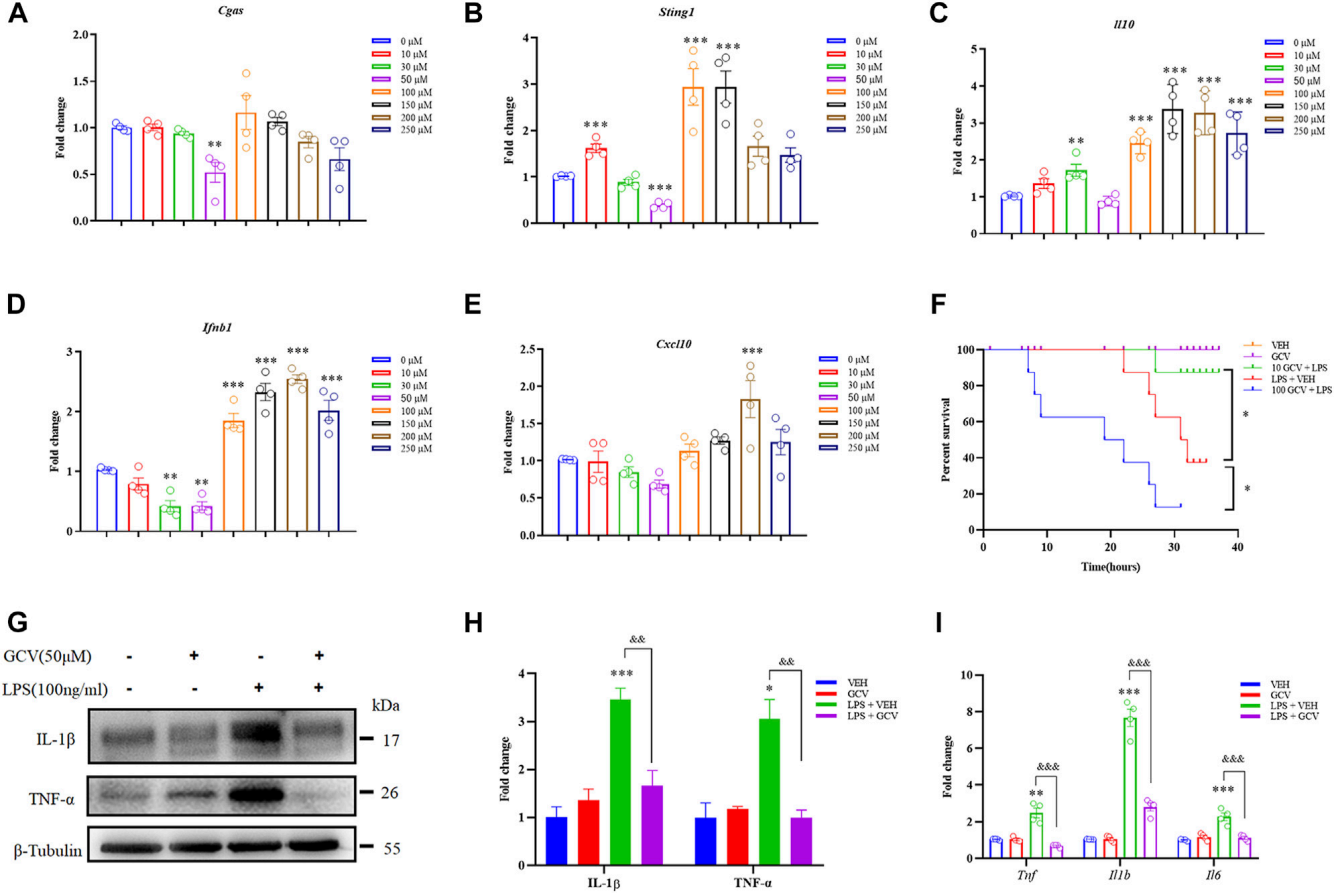
Effects of GCV on expression of inflammatory factors in RAW264.7 cells. **(A–E)** Q-PCR analysis showed dose-dependent effects of GCV treatment for 24 h on the mRNA expression of *Cgas* **(A)**, *Sting1* **(B)**, *Il10* **(C)**, *Ifnb1* **(D)**, and *Cxcl10* **(E)** in RAW264.7 cells. **(F)** Dose-dependent effects of GCV treatment on the mortality associated with LPS-induced sepsis in mice. (*n* = 7-8 per group; ^*^ *p* < 0.05; Log-rank test). **(G)** Western blotting analysis showing the effect of GCV on LPS-induced expression of IL-1β and TNF-α in RAW264.7 cells. **(H)** Statistical analysis results of **(G)**. **(I)** Q-PCR analysis the effect of GCV on LPS-induced mRNA expression of *Il6*, *Il1b*, and *Tnf* in RAW264.7 cells. (*n* = 4 each group, ^*^ *p* < 0.05, ^**^ *p* < 0.01, ^***^ *p* < 0.001 vs. saline; ^&&^ *p* < 0.01, ^&&&^ *p* < 0.001 vs. LPS group, unpaired Student’s *t*-test). All data was expressed as Mean ± SEM. n.s., no significance.

Firstly, we want to address whether and how GCV affect cGAS-STING singling in cultured RAW264.7 cells. The chemical structure of GCV was shown in Supplementary Figure S2. We treated cultured RAW264.7 cells with different concentration of GCV (10-250 μM) for 24 h. In line with previous study (Ding et al., 2014), we also demonstrated that higher dose of GCV (100-250 μM) significantly activated the STING pathway in RAW264.7 cells, reflected by upregulated mRNA expression of *Sting1*, *Il10*, *Ifnb1*, and *Cxcl10* (but not for *Cgas*) (Figures 1A–E). Unexpected, we found that low-dose GCV (50 μM) decreased the mRNA expression levels of *Cgas* (Figure 1A), *Sting1* (Figure 1B) and *Ifnb1* (Figure 1D) in RAW264.7 cells.

In order to determine the effects of different doses of GCV on systemic inflammation *in vivo*, we intraperitoneally (i.p.) injection of mice with lipopolysaccharide (LPS), a component of the cell wall of Gram-negative bacteria (Poltorak et al., 1998). The results showed low-dose GCV (10 mg/kg) significantly improved the survival of mice treated with LPS (*p* < 0.05), but high-dose GCV (100 mg/kg) significantly deteriorated LPS-induced mortality in mice (*p* < 0.05) (Figure 1F). In cultured RAW264.7 cells, Western Blotting analysis showed that LPS induced significant elevation of pro-inflammatory factors (For IL-1β: *p* < 0.001 and for TNF-α: *p* < 0.05) compared with vehicle group. Incubation of low-dose GCV (50 μM) significantly reduced LPS-induced upregulation of IL-1β and TNF-α (For IL-1β: *p* < 0.01 and for TNF-α: *p* < 0.01) (Figures 1G,H). Consistently, Q-PCR analysis also confirmed that LPS-induced up-regulation of mRNA expression of *Il1b*, *Tnf*, and *Il6* was significantly inhibited by GCV (50 μM) (For *Il1b*: *p* < 0.001; For *Tnf*: *p* < 0.001 and For *Il6: p* < 0.001) (Figure 1I).

**FIGURE 2:**
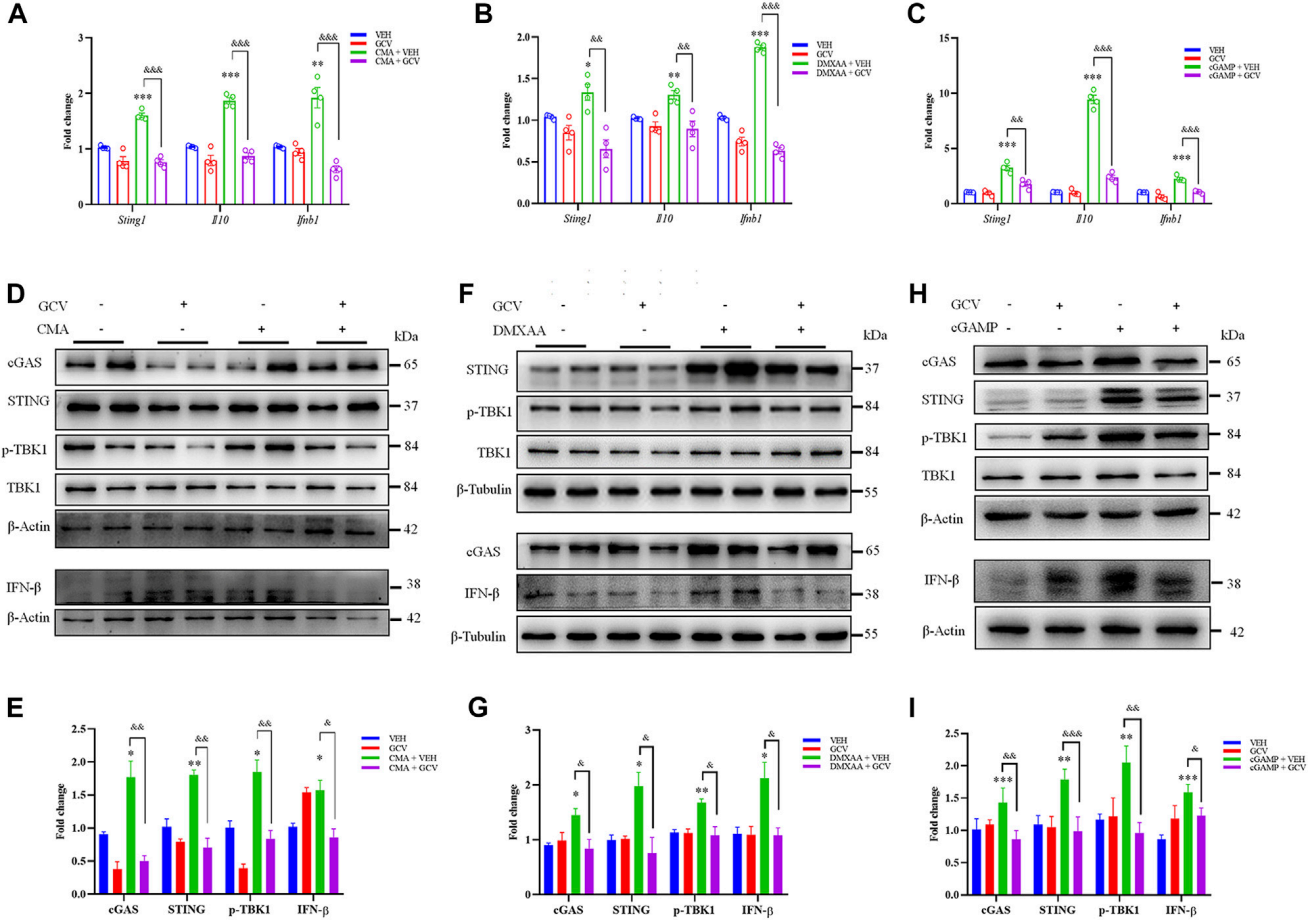
Low-dose GCV inhibited cGAS-STING pathways in RAW264.7 cells. **(A–C)** Q-PCR analysis showed the effect of pretreatment of GCV on up-regulation of mRNA expression of *Sting1*, *Il10*, *Ifnb1* induced by CMA- **(A)**, DMXAA- **(B)**, and cGAMP **(C)** in RAW264.7 cells. **(D)** Western blotting analysis showed the effect of GCV on CMA-induced expression changes of cGAS, STING, IFN-β, and p-TBK1 in RAW264.7 cells. **(E)** Statistical analysis results for **(D)**. **(F)** Western blotting analysis showed the effect of GCV on DMXAA-induced expression changes of cGAS, STING, IFN-β, and p-TBK1 in RAW264.7 cells. **(G)** Statistical analysis results for **(F)**. **(H)** Western blotting analysis showed the effect of GCV on cGAMP-induced expression changes of cGAS, STING, IFN-β, and p-TBK1 in RAW264.7 cells. **(I)** Statistical analysis results for **(H)**. (*n* = 4 each group, ^*^ *p* < 0.05, ^**^ *p* < 0.01, ^***^ *p* < 0.001 vs. saline. ^&^ *p* < 0.05, ^&&^ *p* < 0.01, ^&&&^ *p* < 0.001 vs. STING agonists group, unpaired Student’s *t*-test). (*n* = 4 each group, ^*^ *p* < 0.05, ^**^ *p* < 0.01, ^***^ *p* < 0.001, ^&&^ *p* < 0.01, ^&&&^ *p* < 0.001; unpaired Student’s *t*-test). All data was expressed as Mean ± SEM. VEH, vehicle.

Subsequently, we aimed to explore the effects of low-dose GCV on STING activation caused by selective STING agonists 10-carboxymethyl-9-acridanone (CMA), 5, 6-dimethylxanthenone-4-acetic acid (DMXAA), and 2, 3-cGAMP (an endogenous agonist of STING) in RAW264.7 cells. Q-PCR analysis showed that activation STING by CMA, DMXAA, and cGAMP enhanced the mRNA expression of *Sting1*, *Il10* and *Ifnb1* (For CMA: *Sting1*: *p* < 0.001, *IL10*: *p* < 0.001, and *Ifnb1: p* < 0.01; For DMXAA: *Sting1*: *p* < 0.05, *IL10*: *p* < 0.001, and *Ifnb1: p* < 0.001; For cGAMP: *Sting1*: *p* < 0.001, *IL10*: *p* < 0.001, and *Ifnb1: p* < 0.001) (Figures 2A–C). And GCV (50 μM) treatment abolished all tested STING agonists-induced activation of STING, reflected by decreased mRNA expression levels of *Sting1*, *Il10*, and *Ifnb1* (For CMA: *Sting1*: *p* < 0.001, *IL10*: *p* < 0.001, and *Ifnb1: p* < 0.001; For DMXAA: *Sting1*: *p* < 0.01, *IL10*: *p* < 0.01, and *Ifnb1: p* < 0.001; For cGAMP: *Sting1*: *p* < 0.01, *IL10*: *p* < 0.001, and *Ifnb1: p* < 0.001) (Figures 2A–C). Consistently, Western blotting analysis demonstrated that GCV (50 μM) significantly inhibited these upregulation of protein expression levels of cGAS-STING pathways by all tested STING agonists (For CMA: cGAS: *p* < 0.01, STING: *p* < 0.01, p-TBK1: *p* < 0.01, and IFN-β: *p* < 0.05; For DMXAA: cGAS: *p* < 0.05, STING: *p* < 0.05, p-TBK1: *p* < 0.05, and IFN-β: *p* < 0.05; For cGAMP: cGAS: *p* < 0.01, STING: *p* < 0.001, p-TBK1: *p* < 0.01, and IFN-β: *p* < 0.05) (Figures 2D–I). Thus, the results suggest that low-dose GCV is able to suppress the activation of STING signaling in cultured RAW264.7 cells.

### The cGAS-STING pathways are upregulated in the colon of IBD patients and DSS-induced colitis mice

**FIGURE 3:**
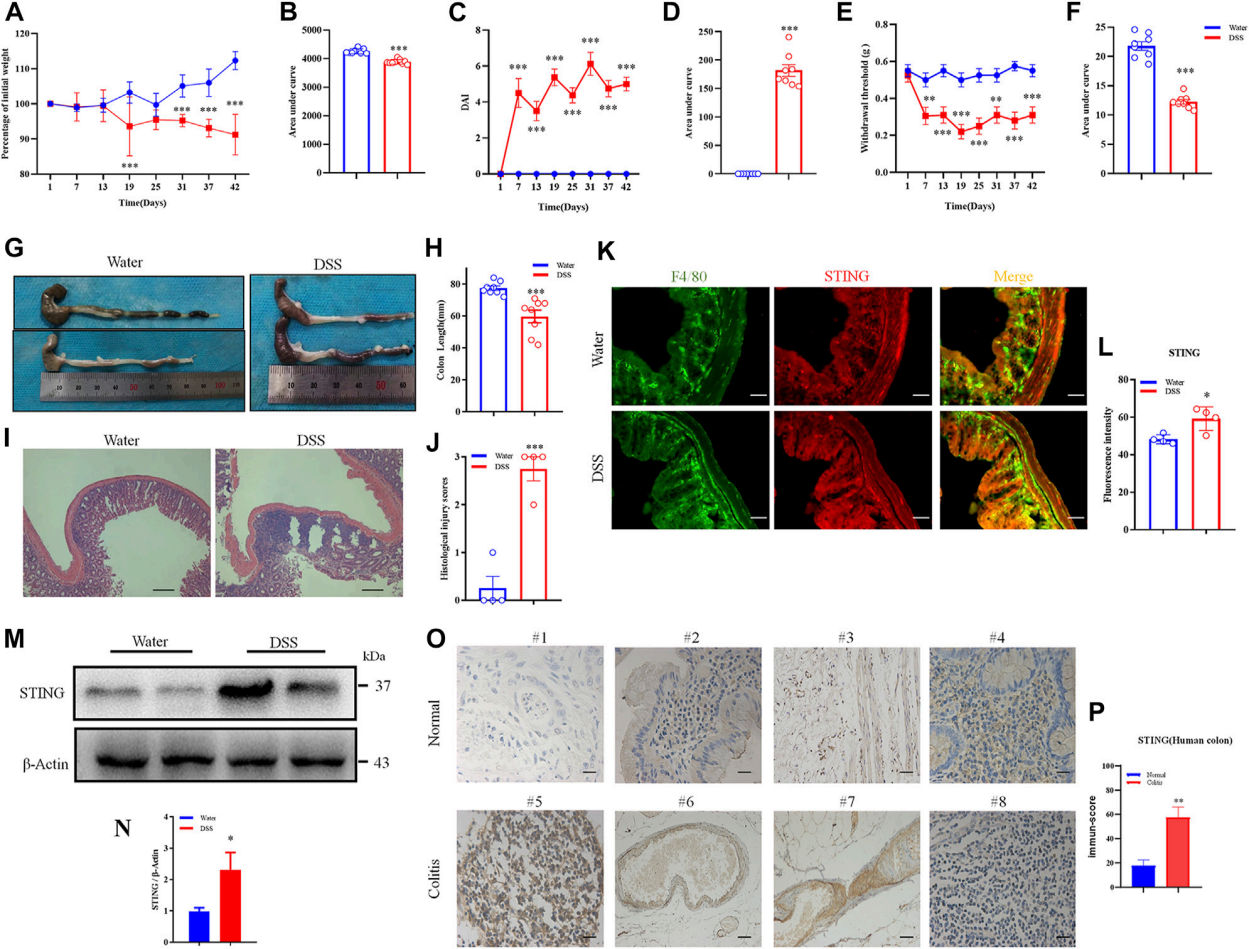
STING was upregulated following DSS-induced chronic colitis in mice and in UC patients. **(A)** Body weight loss was induced by DSS-colitis in mice. **(B)** The quantification of area under the curve (AUC) for **(A)**. **(C)** DSS-colitis induced changes of the Disease activity index (DAI) score. **(D)** The quantification of AUC for **(C)**. **(E)** DSS-colitis induced mechanical pain hypersensitivity in the abdomen. **(F)** The quantification of AUC for **(E)**. (n = 7-8 per group; *p < 0.05, **p < 0.05, ***p < 0.001, DSS vs. vehicle group; two-way ANOVA with post-hoc Bonferroni test). **(G)** Representative pictures showed colon shortening induced by DSS. **(H)** The quantification of colon shortening for **(G)**. (n = 7-8 per group; ***p < 0.001, DSS vs. vehicle group; unpaired Student’s t-test). **(I)** Representative H&E staining of colon sections from DSS group and vehicle group. **(J)** Statistical analysis for **(I)**. (*n* = 4 each group, ***p < 0.001, DSS vs. vehicle group; unpaired Student’s t-test). **(K)** Double immunostaining of STING and F4/80 in the colon tissue from DSS group and vehicle group. **(L)** Statistical analysis for **(K)**, scale bar: 50 μm. (*n* = 4 each group, *p < 0.05, DSS vs. vehicle group; unpaired Student’s t-test). **(M)** Western blotting analysis of STING expression in colon of mice. **(N)** Statistical analysis of Western blotting. (n = 3 each group, *p < 0.05, DSS vs. vehicle group; unpaired Student’s t-test). **(O)** The expression of STING in the colon tissue was increased in patients with ulcerative colitis compared with adjacent tissue of colon cancer. **(P)** Statistical analysis results for **(O)**. (n = 4; **p < 0.01 vs Normal; unpaired Student’s t-test). The scale bar: 20 μm. All data was expressed as Mean ± SEM. DAI, disease activity index; DSS, dextran sulfate sodium.

Subsequently, we determined whether the expression of STING is altered in DSS-induced chronic colitis mouse model and in colitis patients from the Second Affiliated Hospital of Soochow University. In DSS-induced colitis mouse model, clinical parameters were assessed such as body weight loss and disease activity index (DAI) score. DSS-induced colitis mice displayed obvious body weight loss with occurring at the end of cycle two and cycle three compared to control mice (*p* < 0.001) (Figures 3A,B). Increased DAI scores indicated heightened severity of DSS mice compared to control mice (*p* < 0.001) (Figures 3C,D). Abdominal mechanical pain thresholds were significantly reduced in DSS group compared to control mice (*p* < 0.001) (Figures 3E,F). Upon the terminal point of the experiment, macroscopic disease indicators were evaluated such as the pathology of the intestines and colon shortening (Figures 3G,H). DSS-induced colitis mice exhibited colonic shortening compared to control mice (*p* < 0.001) (Figures 3G,H). H&E staining showed that the histopathological injury scores of DSS-induced colitis mice were significantly increased than that of control mice (*p* < 0.001) (Figures 3I,J). Immunofluorescence staining showed that STING expression was up-regulated in the DSS group than that of the control group (*p* < 0.001) (Figures 3K,L). Western blotting analysis also showed that the protein expression level of STING in the colons was elevated in DSS-induced colitis mice (*p* < 0.05) (Figures 3M,N). Clinically, we obtained paraffin sections of adjacent normal tissue of colon cancer and colon tissue of UC patients. Immunohistochemistry analysis showed that the expression of STING in colon tissue was increased significantly in colitis patients compared with the adjacent normal tissues (*p* < 0.01) (Figures 3O,P). The detailed patient information was provided in Supplementary Table S2. Collectively, these result show that up-regulated expression of cGAS-STING pathway was consistently observed in intestinal inflammation in mice and in humans afflicted with UC.

**FIGURE 4:**
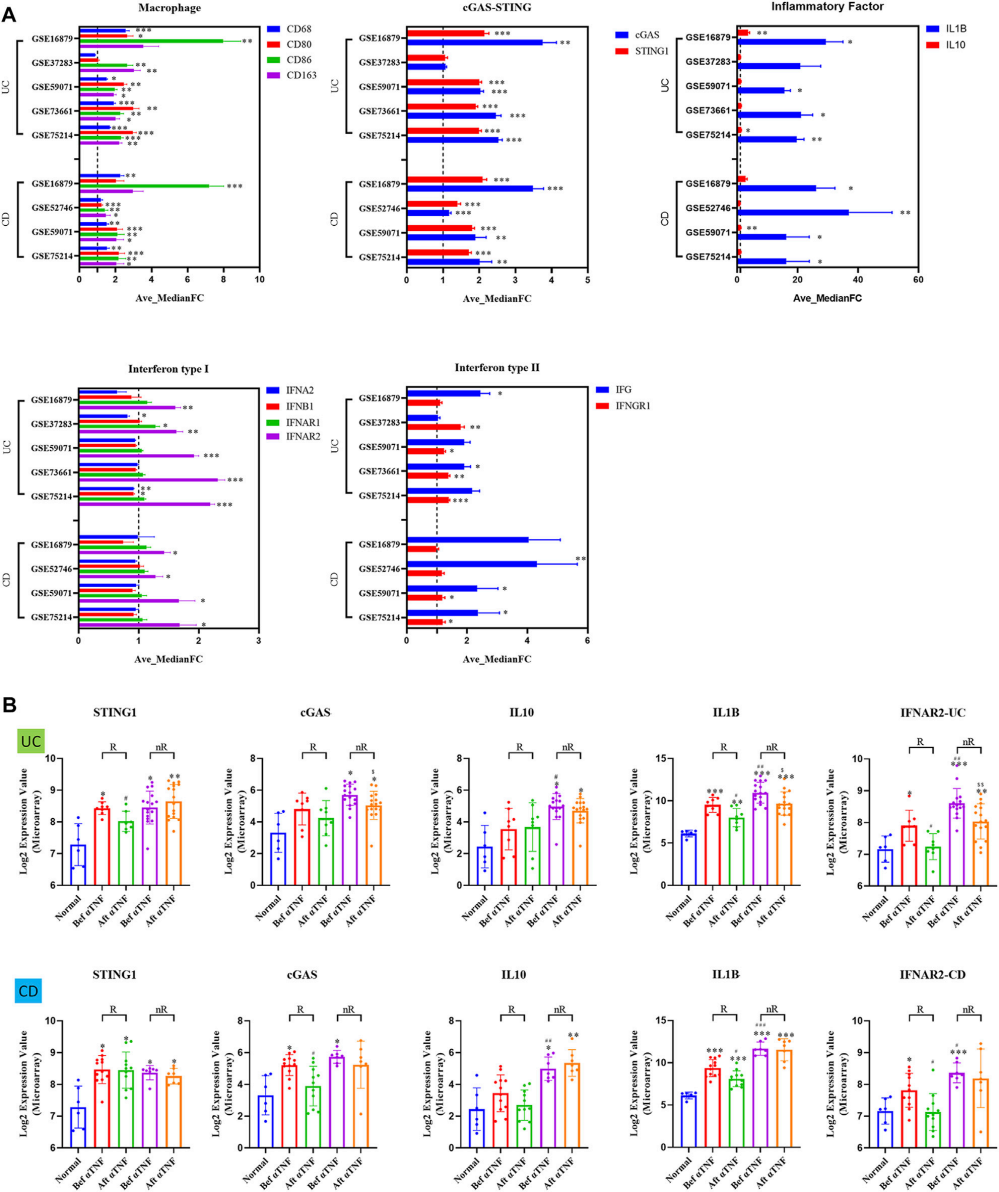
The up-regulation of gene expression of cGAS-STING pathways in IBD patients, including UC and CD. **(A)** The relative expression changes of macrophage cGAS-STING signaling pathway in IBD patients. **(B)** The mRNA levels of cGAS-STING signaling pathway in UC and CD responder/non-responder patients, before and after treatment of anti-TNF (infliximab), compared with non-IBD controls. R., anti-TNF responders; nR., anti-TNF non-responders; Bef αTNF, before anti-TNF treatment; Aft αTNF, after anti-TNF treatment. All data are shown as mean ± SD and are analyzed using one-way ANOVA followed by Dunnett’s multiple comparison test. UC, ulcerative colitis; CD, Crohn’s disease.

With the growing gene expression databases available in the public and the advances of bioinformatics analysis, it is possible to test new hypothesis through screening genes of potential interest. We then performed meta-analysis aimed at clarifying the gene expression changes of cGAS-STING signaling pathway in IBD patients, including UC and CD. A comprehensive analysis of the expression of macrophage markers (such as CD68, CD80, CD86, and CD163) across these studies showed consistent upregulation in both UC and CD patients (Figure 4A). Next, cGAS and STING expression, inflammatory cytokines (IL-1β and IL-10), and type I interferon signaling (only IFNAR2) were all up-regulated across the studies in both UC and CD patients. Additionally, type II interferon signaling (IFN-γ and IFNGR1) were up-regulated across the studies in both UC and CD patients (Figure 4A).

In the abovementioned meta-analysis, we noticed that one study included (GSE16879) focused on anti-TNFα antibody (Infliximab) therapy in IBD patients. Interestingly, we found that αTNF (Infliximab) treatment significantly reduced STING expression in UC patients, but not in CD patients (*p* < 0.05) (Figure 4B). In contrast, αTNF (Infliximab) treatment significantly reduced cGAS expression in CD patients, but not in UC patients (*p* < 0.05) (Figure 1B). Moreover, we demonstrated higher expression in IL-10 (*p* < 0.05), IL-1β (*p* < 0.01), and IFNAR2 (*p* < 0.01) in αTNF (Infliximab) non-responders, compared with responders after αTNF (Infliximab) treatment in UC patients (Figure 4B). In contrast, we demonstrated higher expression in IL-10 (*p* < 0.01), IL-1β (*p* < 0.001), and IFNAR2 (*p* < 0.05) in αTNF (Infliximab) non-responders, compared with responders after αTNF (Infliximab) treatment in CD patients (Figure 4B).

### Low-dose GCV treatment ameliorates DSS-induced colitis in mice

Next, in order to investigate the effect of different doses of GCV on DSS-induced acute colitis *in vivo*, we i.p. injected mice with GCV (10 mg/kg and 100 mg/kg) in DSS-induced colitis in mice. The results demonstrated that high-dose GCV (100 mg/kg) exacerbate DSS-induced acute colitis in mice, but low-dose GCV (10 mg/kg) had clear therapeutic effects on DSS-induced acute colitis, reflected by attenuating DSS-induced weight loss in low-dose GCV treated mice (Supplementary Figure S3).

**FIGURE 5:**
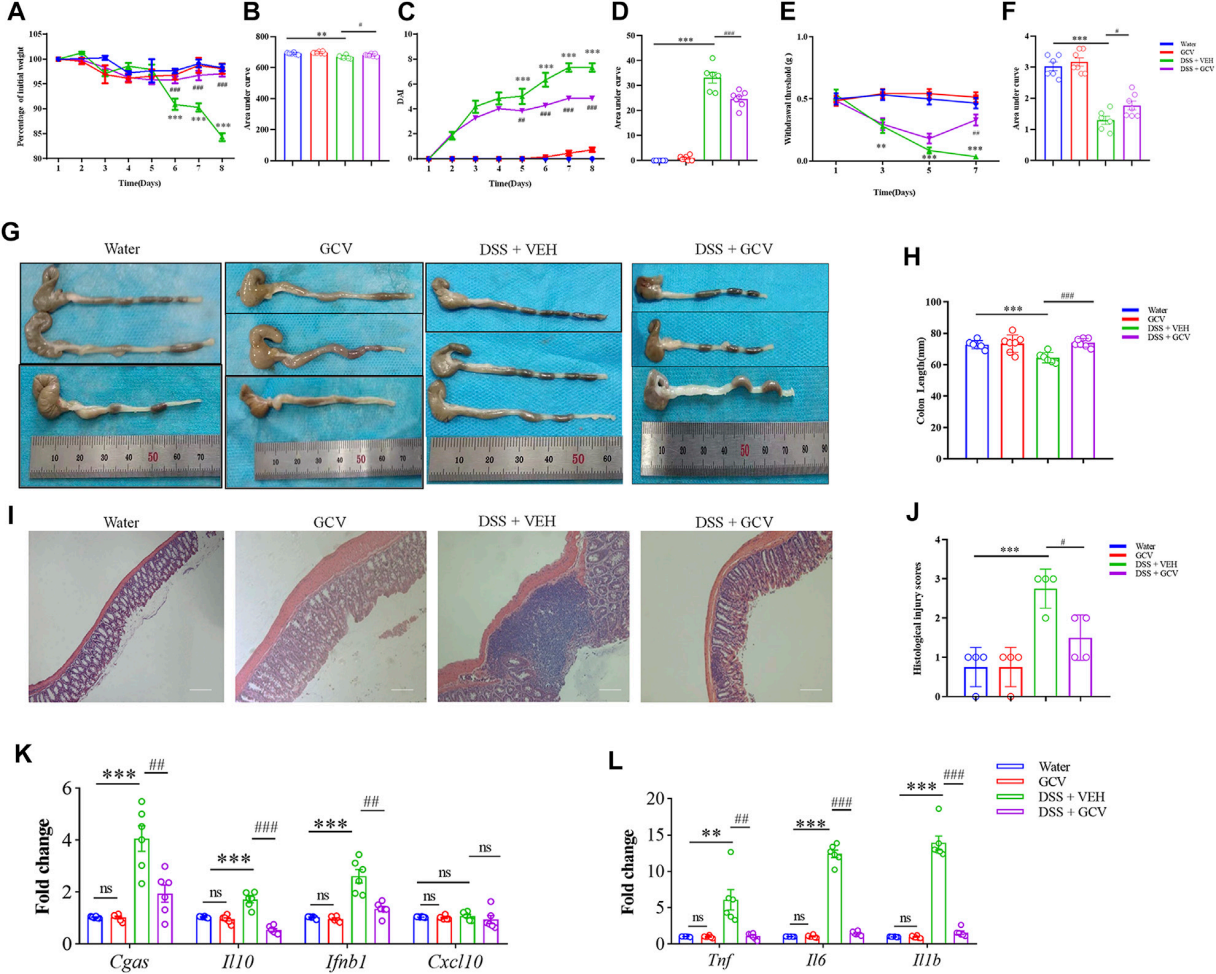
Low-dose GCV attenuated DSS-colitis in mice. **(A)** DSS-colitis induced body weight loss from different groups. **(B)** The quantification of AUC for **(A)**. **(C)** DSS-colitis induced increase of DAI scores in different groups. **(D)** The quantification of AUC for **(C)**. **(E)** DSS-colitis induced mechanical pain hypersensitivity in the abdomen in mice from different groups. **(F)** The quantification of AUC for **(E)**. (*n* = 6-7 per group; ^*^ *p* < 0.05, ^**^ *p* < 0.05, ^***^ *p* < 0.001, DSS vs. vehicle group; ^#^ *p* < 0.05, ^##^ *p* < 0.01, ^###^ *p* < 0.001, GCV + DSS vs. DSS group; ^&^ *p* < 0.05, ^&&^ *p* < 0.01, ^&&&^ *p* < 0.001, STING^gt/gt^ + DSS vs DSS group; two-way ANOVA with post-hoc Bonferroni test). **(G)** Representative pictures of colonic length from different groups. **(H)** Quantification of the colonic length for **(G)**. (*n* = 5-7 each group; ^*^ *p* < 0.05, ^##^ *p* < 0.01, ^&^ *p* < 0.05; one-way ANOVA with post-hoc Bonferroni test) **(I)** Representative photographies of H&E staining of colon sections from different groups. **(J)** Statistical analysis for **(I)** (*n* = 4 each group; ^***^ *p* < 0.001, ^#^ *p* < 0.05, ^&^ *p* < 0.05; one-way ANOVA with post-hoc Bonferroni test). **(K**,**L)** The Q-PCR analysis of mRNA expression of colonic *Cgas*, *Il10*, *Ifnb1*, *Cxcl10*, *Il1b*, *Il6*, and *Tnf* in mice of different group (*n* = 6 per group, ^**^ *p* < 0.01, ^***^ *p* < 0.001, ^##^ *p* < 0.01, ^###^ *p* < 0.001; unpaired Student’s *t*-test). All data was expressed as Mean ± SEM. DSS, dextran sulfate sodium; VEH, vehicle.

Moreover, in order to determine the mechanisms underlying the therapeutic effects of GCV, we chose low-dose GCV (10 mg/kg/day) to perform further investigation *in vivo*. We found that GCV ameliorated DSS-induced body weight loss (*p* < 0.05) (Figures 5A,B), reduced DAI (*p* < 0.001) (Figures 5C,D), and abdominal mechanical pain hypersensitivity (*p* < 0.05) (Figures 5E,F) compared with vehicle group in mice. Analysis of colon morphology showed that GCV treatment ameliorated DSS-induced reduction of colonic length in mice (*p* < 0.001) (Figures 5G,H). Histological analysis by H&E staining in colon sections showed that GCV treatment significantly reduced the histological injury scores in DSS-induced colitis mice compared with vehicle group (Figures 5I,J). Q-PCR analysis showed that the GCV treatment markedly inhibited DSS-induced up-regulation of the mRNA expression levels of *Cgas* (*p* < 0.01), *Il10* (*p* < 0.001), *Ifnb1* (*p* < 0.01), *Tnf* (*p* < 0.01), *Il6* (*p* < 0.001), and *Il1b* (*p* < 0.001) in colon of mice (Figures 5K,L).

**FIGURE 6:**
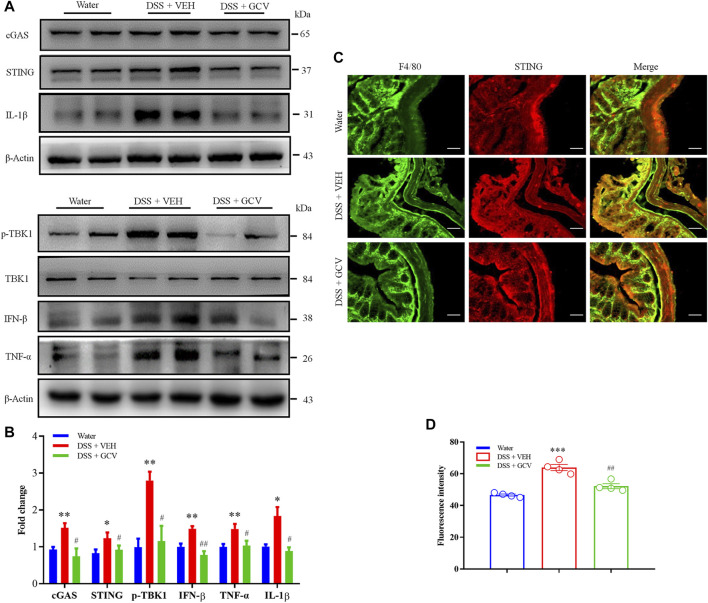
Low-dose GCV attenuated cGAS-STING pathways in the colon of DSS-colitis in mice. **(A)** Western blotting analysis showed that protein expression of colonic STING, cGAS, p-TBK1, IFN-β, IL-1β, and TNF-α in mice of different treatment group. **(B)** Statistical analysis for **(A)** (*n* = 4 per group, ^*^ *p* < 0.05, ^**^ *p* < 0.01, DSS vs. vehicle group; ^#^ *p* < 0.05, ^##^ *p* < 0.01, ^###^ *p* < 0.001, GCV + DSS vs. DSS group; unpaired Student’s *t*-test). **(C)** Double immunostaining of STING and F4/80 in the colon tissue. **(D)** Statistical results for **(C)**. Scale bar = 50 μm. (*n* = 4 each group, ^***^ *p* < 0.001, ^##^ *p* < 0.01; unpaired Student’s *t*-test). All data was expressed as Mean ± SEM.

Subsequently, we investigated the effects of GCV on the up-regulated expression of cGAS-STING pathways in DSS-induced colitis in mice. Western blotting analysis showed that GCV treatment significantly attenuated DSS-induced up-regulation of protein expression of cGAS (*p* < 0.05), STING (*p* < 0.05), p-TBK1 (*p* < 0.05), IL-1β (*p* < 0.05), TNF-α (*p* < 0.05), and IFN-β (*p* < 0.01) in the colon in mice (Figures 6A,B). Immunofluorescence analysis showed that STING was mainly co-localized with a macrophage marker F4/80 in the colon of native mice (Figure 6C). On 8 days after DSS treatment, we demonstrated significant upregulation of expression of STING and F4/80 in the macrophages in the colon tissue in mice (Figure 6C). In addition, the GCV treatment significantly suppressed DSS-induced up-regulation of STING expression in mice (*p* < 0.01) (Figures 6C,D). Collectively, these results suggest that GCV treatment inhibits DSS-induced enhanced expression of cGAS-STING signaling in the colon in mice.

### DSS-induced colitis is attenuated in STING deficient mice

**FIGURE 7:**
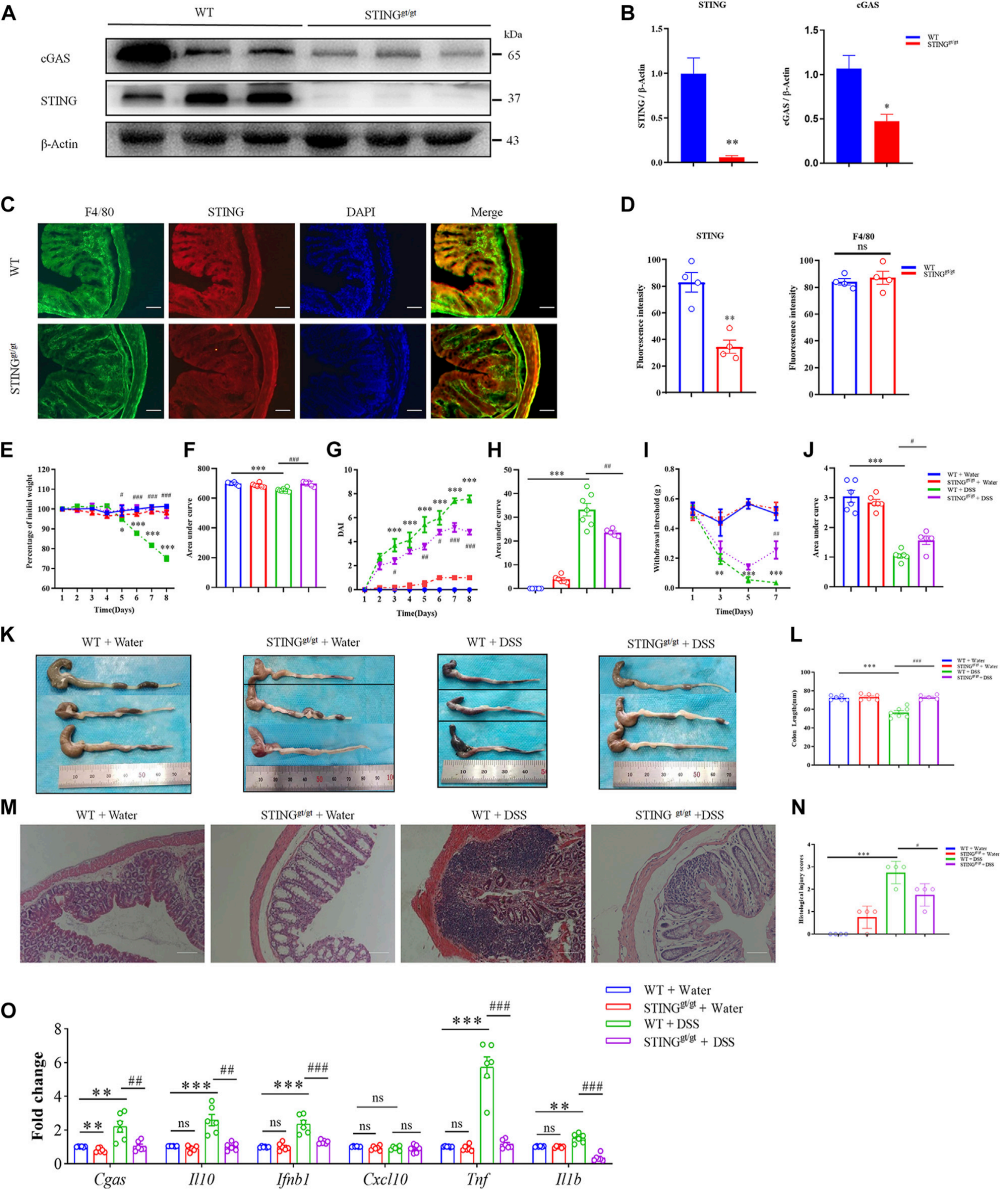
STING deficiency attenuated DSS-colitis in mice. **(A)** Representative Western blotting detected cGAS and STING expression in STING^gt/gt^ and WT mice. **(B)** Statistical analysis for **(A)**. **(C)** Representative immunofluorescence detected STING and F4/80 expression in STING^gt/gt^ and WT mice. **(D)** Statistical analysis for **(C)**. (*n* = 3 each group, ^**^ *p* < 0.01, STING^gt/gt^ vs. vehicle group; unpaired Student’s *t*-test). Wild-type (WT) and STING^gt/gt^ mice were given 3% dextran sulfate sodium (DSS) in drinking water for 8 days. **(E)** Body weight changed following DSS administration in mice. **(F)** The quantification of AUC for **(E)**. **(G)** DSS-induced changes of DAI score in WT and STING^gt/gt^ mice. **(H)** The quantification of AUC for **(G)**. **(I)** DSS-induced changes of mechanical pain sensitivity in the abdomen in STING^gt/gt^ mice and WT mice. **(J)** The quantification of AUC for **(I)**. (*n* = 5-7 per group; ^**^ *p* < 0.01, ^***^ *p* < 0.001, DSS vs. vehicle group; ^#^ *p* < 0.05, ^##^ *p* < 0.01, ^###^ *p* < 0.001, STING^gt/gt^ + DSS vs DSS group; two-way ANOVA with post-hoc Bonferroni test). **(K)** Representative pictures of colons from WT and STING^gt/gt^ mice on day 8. **(L)** Quantification of the colon length in **(K)**. (*n* = 5-7 per group; ^***^ *p* < 0.001, ^###^ *p* < 0.001; unpaired Student’s *t*-test). **(M)** Representative photographies of H&E staining of colon sections from 4 different groups. **(N)** Statistical analysis for **(M)** (*n* = 4 each group; ^***^ *p* < 0.001, ^#^ *p* < 0.05; unpaired Student’s *t*-test). **(O)** The Q-PCR analysis of mRNA expression of colonic *Cgas*, *Il10*, *Ifnb1*, *Cxcl10*, *Il1b,* and *Tnf* in mice from different group (*n* = 6 each group, ^**^ *p* < 0.01, ^***^ *p* < 0.001, ^#^ *p* < 0.05, ^##^ *p* < 0.01, ^###^ *p* < 0.001; unpaired Student’s *t*-test). All data was expressed as Mean ± SEM. DSS, dextran sulfate sodium; n.s., no significance. WT, wild type.

Then, we employed STING deficient mice (STING^gt/gt^ mice) to further determine the functional effects of STING on the pathogenesis of DSS-induced colitis in mice. Western blotting analysis confirmed the absence of STING expression in the colon in STING^gt/gt^ mice (*p* < 0.01) (Figures 7A,B). Moreover, the protein expression of cGAS in the colon was also significantly decreased in STING deficient mice compared with WT mice (*p* < 0.05) (Figures 7A,B). Immunofluorescence analysis verified that diminished STING expression in STING^gt/gt^ mice (*p* < 0.01) (Figures 7C,D). However, the expression of macrophage marker F4/80 was not affected in STING^gt/gt^ mice (Figures 7C,D). In order to evaluate the effects of STING deficiency on the development of DSS-induced colitis in mice, mice were given 3% DSS drinking water for 8 days to induce acute colitis in WT and STING^gt/gt^ mice. The results showed that STING^gt/gt^ mice exhibited markedly decreased disease severity compared with WT mice during DSS-induced colitis as measured by weight loss (*p* < 0.001) (Figures 7E,F) and disease activity index (DAI) (*p* < 0.01) (Figures 7G,H). Moreover, DSS-induced abdominal mechanical pain hypersensitivity was relieved in STING deficient mice (*p* < 0.05) (Figures 7I,J). Histological analysis showed that DSS-induced colonic length shorten was significantly inhibited in STING^gt/gt^ mice (*p* < 0.001) (Figures 7K,L). Next, H&E staining analysis showed colon sections from STING^gt/gt^ mouse showed an intact epithelium, well-defined crypt length, edema in mucosa and submucosa, and no ulcers or erosions compared with that of WT mice (Figure 7M). Additionally, DSS-induced enhanced histological injury scores was ameliorated in STING^gt/gt^ mice compared with that of WT mice (*p* < 0.05) (Figure 7N).

We investigated the mRNA expression changes of cGAS-STING pathway in the WT mice and STING^gt/gt^ mice. Q-PCR analysis showed that DSS-colitis-induced up-regulated mRNA expression of *Cgas*, *Il10*, *Ifnb1*, *Tnf*, and *Il1b* was significantly reduced in STING deficient mice (For *Cgas*: *p* < 0.01; For *Il10*: *p* < 0.01; For *Ifnb1*: *p* < 0.001; For *Tnf*: *p* < 0.001; For *Il1b*: *p* < 0.001) (Figure 7O).

### STING deficient mice are protected from DSS-induced dysbiosis

To better understand the role of the STING in the regulation of microbiota composition (Canesso et al., 2018), we evaluated the differences in the composition of the gut microbiota in four groups, including WT + Water group, WT + DSS group, STING^gt/gt^ + Water, and STING^gt/gt^ + DSS. Overall, the 16S rRNA gene sequencing yielded 46044 to 67208 valid tags with average lengths ranging from 409.18 to 418.76 bp. In addition, 9812 OTUs (Operational Taxonomic Units) were identified with a 97% similarity cutoff.

To validate the adequacy of these selected samples, we compared species accumulation curves among all the samples. We found when the sample size was greater than 20, the curve was flat, and the species in this environment no longer increased with the increase of the sample size. It indicates the sequencing samples is adequate (Supplementary Figure S4A). We compared rarefaction curve among all the samples. When the curve flattens with the increase of the number of extracted sequences, it indicates that the amount of sequencing data in the sample is reasonable (Supplementary Figure S4B). The beta diversity using principal coordinate analysis (PCA) plots based on the unweighted and weighted UniFrac distance. The β-diversity analysis revealed a distinct clustering of the bacterial communities in the DSS group compared with water-treated mice. However, in STING^gt/gt^ group and STING^gt/gt^ + DSS group, using the same β-diversity matrix, we found no difference in the bacterial profiles (Supplementary Figures S5A,B). To evaluate the differences in bacterial diversity among all the groups, sequences were aligned to estimate alpha diversity using Chao 1 and observed index. Both Chao1 estimators and observed index were significantly decreased in the WT + DSS group, STING^gt/gt^ group, STING^gt/gt^ + DSS group compared with the control group, indicating a lower richness and evenness of gut bacteria in these groups. Interestingly, WT + Water group and WT + DSS group had differences in Chao1 estimators and observed index. STING^gt/gt^ + Water group and STING^gt/gt^ + DSS group had no differences for Chao1 estimators and observed index (Supplementary Figure S6). Thus, the results reveal that STING deficiency prevents species richness changes in DSS-induced intestinal inflammation.

**FIGURE 8:**
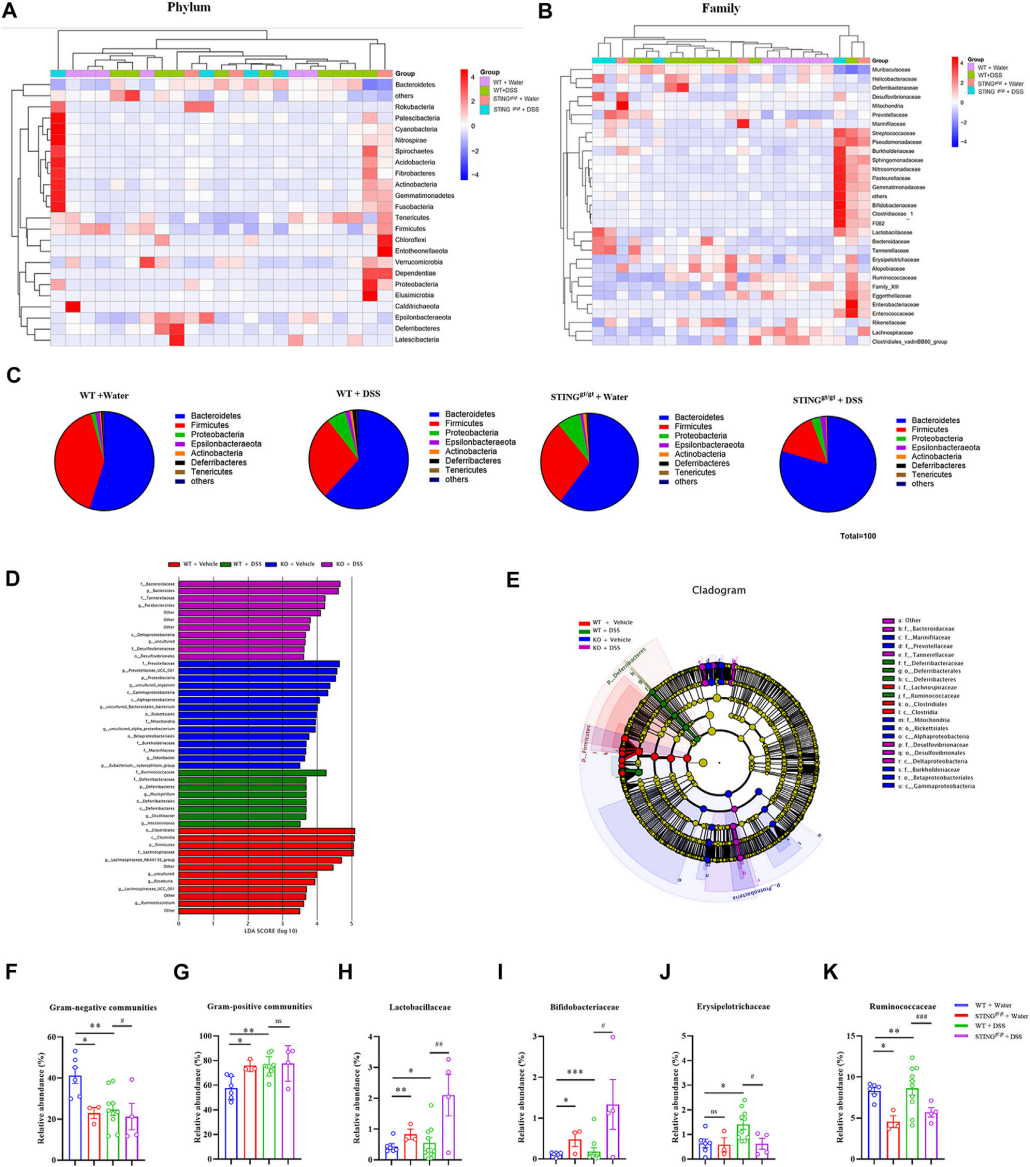
STING deficiency prevented dysbiosis induced by DSS-colitis in mice. **(A)** Heatmap analyses of differentially abundant phylum between the vehicle group, WT + DSS group, vehicle + STING^gt/gt^ group, STING^gt/gt^ + DSS groups. **(B)** Heatmap analyses of relative abundant bacterial family in each four group. **(C)** Relative abundance of the main bacterial phylum in different group. **(D)** LEfSe score plot of the discriminative microbial taxa that are more enriched in the vehicle group (red), DSS (green), STING^gt/gt^ (blue), and STING^gt/gt^ + DSS(violet) groups. **(E)** Identification of differentially abundant microbial taxa using linear discriminant analysis effect size (LEfSe) analysis. LEfSe cladogram of the discriminative microbial taxa; the size of the circle showed the relative abundance of the taxa, and the colour shows the different group (red, vehicle group; green, DSS group; blue, STING^gt/gt^ group; violet, STING^gt/gt^ + DSS group). **(F,G)** Quantification of the relative abundance of Gram-negative communities **(F)** and Gram-positive communities **(G)** from **(A)**. **(H–K)** Quantification of the relative abundance of Lactobacillales **(H)**, Bifidobacteriales **(I)**, Bacteroidaceae **(J)**, Ruminococcaceae **(K)** from **(B)**. (*n* = 3–10 each group, n.s., no significance, ^*^ *p* < 0.05, ^**^ *p* < 0.01, ^***^ *p* < 0.001, unpaired Student’s *t*-test). All data was expressed as Mean ± SEM. WT, wild type. DSS, dextran sulfate sodium; WT, wild type.

Then, we obtained the relative abundance of the top abundant microbial taxa at the phylum level and family level to produce a heat map (Figures 8A,B). We detected Proteobacteria phylum overgrowth in STING^gt/gt^ mice, which is related with development of gut inflammation, which is in line with previous report (Canesso et al., 2018). But we also found that there was a lower relative abundance in Proteobacteria phylum in the STING^gt/gt^ + DSS group compared with DSS group (Figure 8C). To further investigate the key phenotypes contributing to the differences among the groups, we performed the LEfSe analysis. According to the LDA score, the optimal-enriched taxa in the stool microbiome of the control group were *Clostridiales, Clostridia, Firmicutes, f_Lachnospiraceae*. The DSS group displayed higher abundance of *Ruminococcaceae, Deferribacteraceae, Muscispirillum*. The STING^gt/gt^ group showed rich abundance of *Prevotellaceae, Prevotellaceae_UCG_001, Proteobacteria,* and *Cammaproteobacteria*. Whereas *Bacteroidaceae, Bacteroides, Tannerellaceae,* and *Parabacteroides* were rich abundant in the STING^gt/gt^ + DSS group (Figure 8D). To continuously observe major flora changes and linkages from the phylum level to the species level, we used the LEfSe to generate a cladogram to identify the major bacteria difference among all four group (Figure 8E). We observed the relative abundance of Gram-negative bacteria was decreased in STING^gt/gt^ mice (*p* < 0.05) (Figures 8F,G), which are frequently associated with IBD in humans and in murine models of intestinal inflammation (Shmuel-Galia et al., 2021). Additionally, compared with the WT + DSS group, the abundance of *Lactobacillaceae* (*p* < 0.01) and *Bifidobacteriaceae* (*p* < 0.05) at the family level was increased in the STING^gt/gt^ + DSS group, while the abundance of *Erysipelotrichaceae* (*p* < 0.05) and *Ruminococcaceae* (*p* < 0.001) was decreased. Thus, these data indicates that DSS-induced dysbiosis may be attenuated in STING deficiency mice (Figures 8H–K).

### Low-dose GCV lacks therapeutic effects on DSS-colitis in STING deficient mice

**FIGURE 9:**
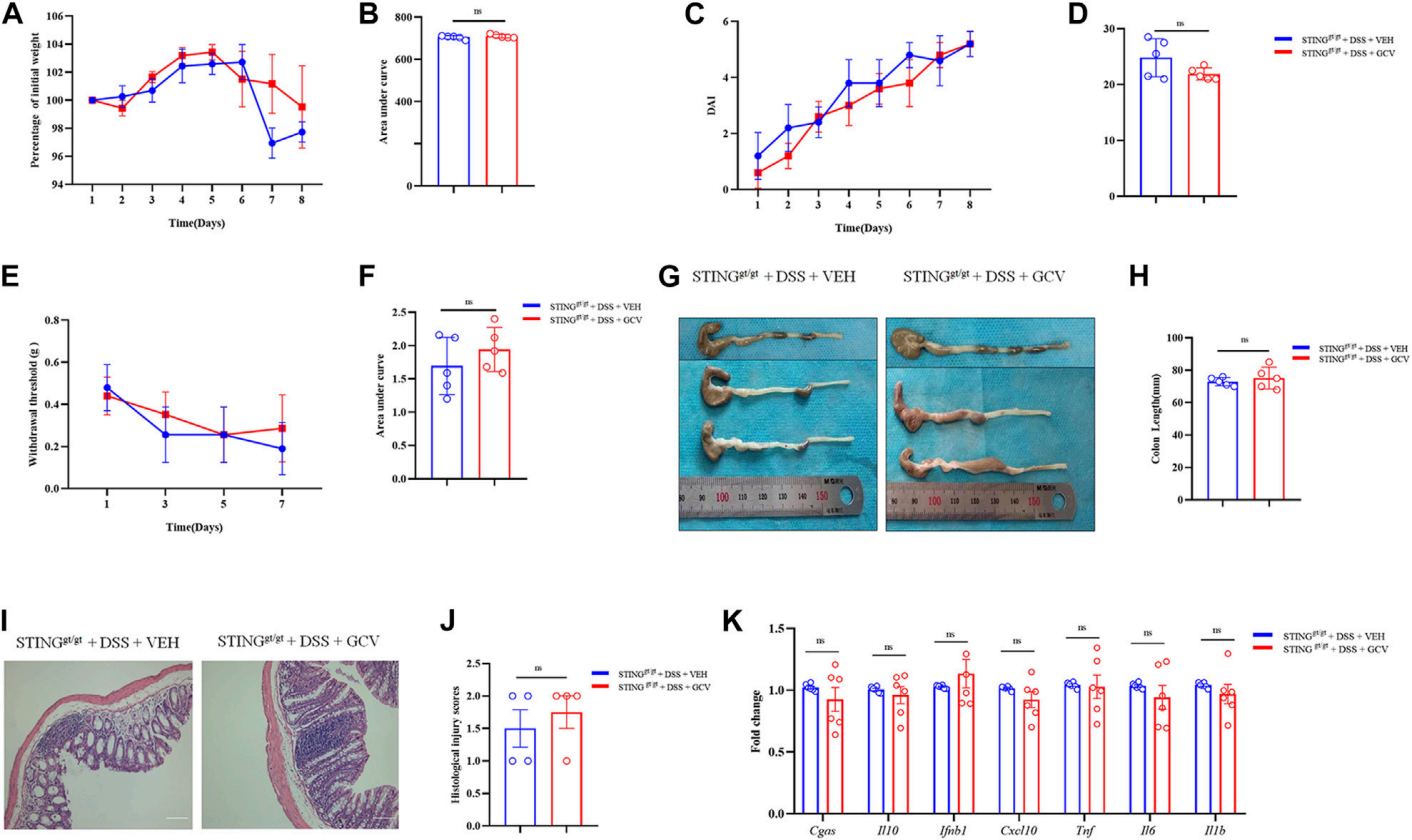
Low-dose GCV had no therapeutic effects on DSS-colitis in mice. **(A)** DSS-colitis induced body weight changes in different groups. **(B)** The quantification of AUC for **(A)**. **(C)** DSS-colitis induced DAI score changes of mice in different groups. **(D)** The quantification of AUC for **(C)**. **(E)** DSS-colitis induced mechanical pain hypersensitivity in the abdomen in mice from different groups. **(F)** The quantification of AUC for **(E)**. (*n* = 5-7 per group; n.s., no significance, ^#^ *p* < 0.05, ^###^ *p* < 0.001, GCV + DSS vs. DSS group; ^&^ *p* < 0.05, ^&&&^ *p* < 0.001, STING^gt/gt^ + DSS vs DSS group; two-way ANOVA with post-hoc Bonferroni test). **(G)** Representative pictures of colons from different groups. **(H)** Quantification of the colonic length for **(G)**. (*n* = 5-7 each group; ^##^ *p* < 0.01; ^###^ *p* < 0.001; unpaired Student’s *t*-test). **(I)** Representative photographies of H&E staining of colon sections from different groups. **(J)** Statistical results for **(I)** (*n* = 4 each group; ^#^ *p* < 0.05; unpaired Student’s *t*-test). **(K)** Q-PCR analysis showed the mRNA expression changes of *Cgas*, *Il10*, *Cxcl10*, *Ifnb1*, *Tnf*, *Il6*, and *Il1b* from different group (*n* = 6 per group, unpaired Student’s *t*-test). All data was expressed as Mean ± SEM. n.s., no significance.

Finally, we employed STING^gt/gt^ mice to determine the role of STING in the therapeutic effects of low-dose GCV in DSS-induced colitis. We found that GCV treatment (10 mg/kg/day) did not affect DSS-disease severity in STING^gt/gt^ mice, reflected by lack effects of GCV on DSS-induced weight loss (*p* > 0.05) (Figures 9A,B) and increased DAI (*p* > 0.05) in STING deficient mice (Figures 9C,D). Compared with WT mice, GCV treatment had no obvious effects on abdominal mechanical pain hypersensitivity in DSS-treated STING deficient mice (*p* > 0.05) (Figures 9E,F). Analysis of colon morphology showed that low-dose GCV treatment had no obvious effects on colonic shorting in DSS-treated STING deficient mice (*p* > 0.05) (Figures 9G,H). Next, we assessed the intestine pathology and examined the H&E staining in the colon sections. When comparing sections of STING^gt/gt^ + DSS group and STING^gt/gt^ + DSS + GCV group mice, we did not observe any obvious colonic mucosal difference, reflected by similar histological injury scores between two groups (*p* > 0.05) (Figures 9I,J). We finally investigated the effects of GCV on the expression levels of cGAS-STING pathways induced by DSS-colitis in STING^gt/gt^ mice. The results showed that the mRNA expression levels of *Cgas*, *Il10*, *Cxcl10*, *Ifnb1*, *Tnf*, *Il6*, and *Il1b* exhibited no obvious difference between GCV and vehicle treatment groups in DSS-colitis in STING^gt/gt^ mice (all *p* > 0.05) (Figure 9K). Therefore, low-dose GCV lacks therapeutic effects on DSS-colitis in STING deficient mice.

## Discussion

GCV as a prodrug nucleoside analogue was developed in the 1970s as an antiviral treatment, which in its canonical function requires bioactivation by viral thymidine kinase (tk) from viruses of the Herpesviridae family, such as cytomegalovirus, Epstein-Barr virus, or HSV (Matthews and Boehme, 1988; Faulds and Heel, 1990). Today, GCV treatment remains the drug of choice for the prevention and treatment of cytomegalovirus (CMV) infection in transplant recipients (Scott et al., 2004). Besides its potent effects on viral replication, GCV at therapeutic concentrations also inhibits proliferation of uninfected cells, most notably of bone marrow cells, possibly through other unknown mechanisms (Ding et al., 2014). Notably, it was recently demonstrated that the type I interferon response in microglia induced by GCV was not mediated through STING, attributing to the ability of GCV to reduce neuroinflammation in the cultured microglia and in a mouse model of multiple sclerosis (experimental autoimmune encephalomyelitis, EAE) (Mathur et al., 2017). Thus, repurposing anti-viral drug GCV may be a promising strategy for anti-inflammatory therapy.

STING is an ER adaptor that recognizes cGAMP and triggers innate immune activation, which has important functions in infection, inflammation and cancer (Barber, 2015). STING was mainly expressed in macrophages in diverse tissues, such as liver and intestine. Activation of STING signaling pathway in macrophages enhanced inflammatory responses (Wang et al., 2020). In the present study, we first explored the possible effects of GCV on STING signaling pathways. We cultured RAW264.7 cells to examine the effects of GCV on pro-inflammatory responses, especially associated with STING activation. We found that low-dose GCV (50 μM) inhibited STING agonists-induced activation of STING signaling in RAW264.7 cells. Moreover, high-dose GCV activated STING signaling, in line with previous findings that high-dose GCV activated STING signaling in cultured BV-2 cells (Mathur et al., 2017). Based on structural similarity between GCV and cGAMP, we postulated that GCV may act as a STING partial agonist. Together, we identified low-dose GCV may be able to inhibit STING pathway in macrophages.

In the present study, we further explored that possible therapeutic effects of GCV on DSS-induced colitis in mice. Oral administration of DSS to mouse leads to clinical symptoms and histopathological features similar to those are observed in UC patients, which was widely used to investigate mechanisms and the therapeutic effects of drug candidates (Wirtz and Neurath, 2007; Yan et al., 2018). DSS-colitis induced epithelial barrier dysfunction, cytokine dysregulation, and mucosal pathology (Elson et al., 1995), which is suitable to study the contribution of innate immune cells to colitis (Dieleman et al., 1994; Williams et al., 2001). After DSS-induced gut injury, large numbers of macrophages are recruited to colon tissues from IBD patients and animal models (Rugtveit et al., 1994; Schenk et al., 2007; Ji et al., 2016). In the present study, we identified that low-dose GCV (10 mg/kg) reduced the DSS-colitis severity in mice, but not for high-dose GCV (100 mg/kg). Consistent with these findings, low-dose GCV (10 mg/kg) protected mice from LPS-induced sepsis in mice, but high-dose GCV (100 mg/kg) oppositely exacerbated LPS-induced mortality. Moreover, DSS-induced up-regulation of cGAS-STING signaling in the colon tissues was attenuated by low-dose GCV in mice. Finally, we determined that low-dose GCV lacks therapeutic effects on DSS-colitis in STING deficient mice, indicating STING mediates the therapeutic effects of low-dose GCV on colitis. In clinical practice, cytomegalovirus (CMV) infection is relative common in IBD patients and is still a complicated problem (Ko et al., 2015). CMV infection has harmful effects in this situation, and it may be related to the decreased response to steroids and other immunosuppressive agents (Pillet et al., 2016). In refractory UC, it is now recommended to use GCV in CMV-infected UC patients (Pillet et al., 2016). Thus, therapeutic effects of GCV in UC warrant further investigation.

STING is found to be a key adaptor mediating innate immune signaling pathway to exogenous or endogenous DNA (Zhang et al., 2020). Recently, STING is rapidly emerging as a critical regulator of intestinal homeostasis (Zhang et al., 2020). Because of the ability of STING activation to drive the production of type I interferons and pro-inflammatory cytokines, it is tightly regulated to maintain intestinal homeostasis (Ke et al., 2022). However, the role of STING in intestinal inflammation remains controversial. Previous early report demonstrated that STING has a protective effect in maintaining the stability of the intestinal environment and controlling intestinal inflammation (Canesso et al., 2018). Consistently, STING-deficient mice are susceptible to DSS-induced colitis in mice (Zhu et al., 2014; Canesso et al., 2018). In addition, it was reported that STING-deficient mice were also highly susceptible to the development of colitis-induced colorectal cancer (Zhu et al., 2014). STING-deficient mice have reduced resistance to the induced epithelial cancer caused by inflammation (Ahn et al., 2014). Conversely, there are much evidence supporting STING over-activation is linked to the exacerbation of colitis. STING-deficient mice were rescued from IL-10 deficiency-induced spontaneous colitis in mice (Ahn et al., 2017). Transgenic mice bearing an allele of constitutively active STING developed spontaneous colitis and gut dysbiosis (Shmuel-Galia et al., 2021). Consistently, pharmacological activation of STING by a STING agonist exacerbated DSS-induced experimental colitis in mice (Martin et al., 2019). It was also found that STING expression was increased in liver and promoted macrophage-mediating hepatic inflammation in patients with non-alcoholic fatty liver disease (NAFLD) (Luo et al., 2018). Moreover, STING deficient mice developed less severe acute pancreatitis and administration of a STING agonist deteriorated acute pancreatitis (Zhao et al., 2018). Thus, given these conflict reports, the roles of STING signaling in the control of inflammation remain unclear.

In the present study, we re-examined the role of STNG in intestinal inflammation by using STING^gt/gt^ mice, a widely used STING deficient mice (Donnelly et al., 2021). Our results demonstrated that STING deficient mice exhibited reduced DSS-induced colitis, including attenuated colon shortening, reduced weight loss, less abdominal mechanical pain, and decreased DAI of colon tissues compared with that of WT mice. Additionally, DSS-colitis associated gut dysbiosis was also improved in STING deficient mice. In support of our findings, previous study also showed that the severity of DSS-colitis was marked exacerbated by a STING agonist in mice (Martin et al., 2019). Collectively, our data supported the notion that excessive activation of STING signaling may play a critical role in the pathogenesis of DSS-induced colitis in mice.

However, there are several possible drawbacks in our study. First, epidemiological incidence and prevalence among different age groups in IBD demonstrated significant sex-based differences, and men and women develop distinct clinical symptoms and disparity in severity of disease. However, our current study only used male mice. Thus, studies taking into account this sexual bias were warranted. Second, although we identified that low-dose GCV has therapeutic effects on DSS-induced colitis, the detailed dose-response curve of GCV need further experiments. Third, the role of gut microbiota in the therapeutic effects of GCV were not evaluated. Follow up research to address these issues is thus required.

## Conclusion

**FIGURE 10:**
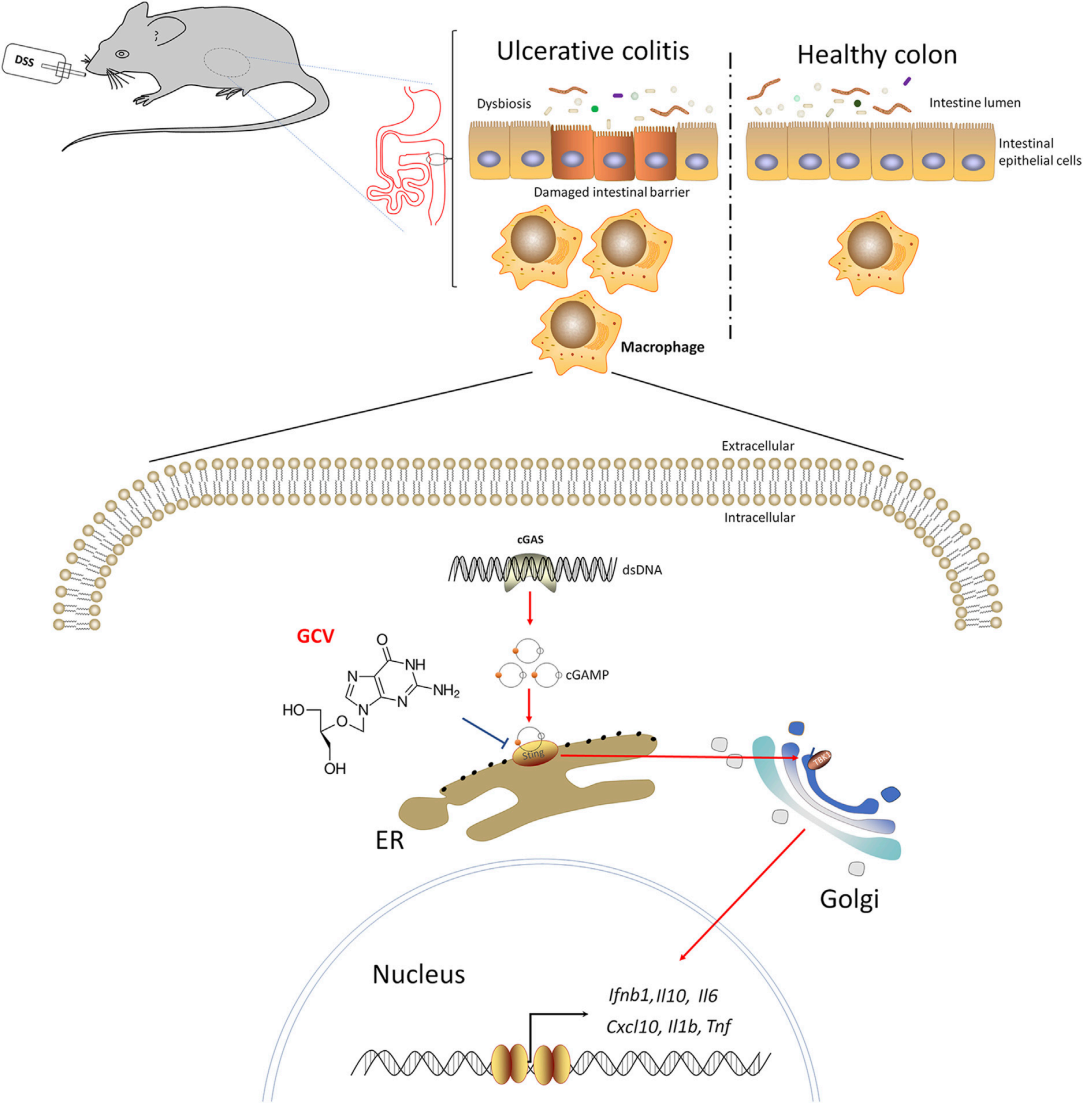
The working hypothesis of this study.

Discovering novel application for known drug (namely drug repurposing) is considered as an effective strategy to reduce the cost and risk of drug development for UC treatment. We find that low-dose GCV may act as a novel pharmacological inhibitor of STING signaling to alleviate DSS-induced colitis in mice. Moreover, cGAS-STING pathways were upregulated in DSS-colitis mice and in UC patients and the severity of DSS-colitis and dysbiosis were attenuated in STING deficient mice. The working hypothesis was shown in Figure 10. Given GCV is a FDA-approved drug, thus repurposing GCV for the treatment of UC warrants further investigation.

## Data Availability

The data that support the findings of this study are available from the corresponding authors upon reasonable request.

## Abbreviations

CDNs, cyclic dinucleotides; CMV, cytomegalovirus; CMA, 9-Oxo-10(9H)-acridineacetic acid; cGAMP, cyclic GMP–AMP synthase; Ctrl, control; CD, Crohn’s disease; DDX41, DEAD-box helicase 41; DSS, dextran sodium sulfate; DAI, disease activity index; DMXAA, 5, 6-dimethylxanthenone-4-acetic acid; EAE, experimental autoimmune brain Myelitis; ER, endoplasmic reticulum; GCV, ganciclovir; HSC, hepatic stellate cell; HSV, Human herpesvirus; IBD, inflammatory bowel disease; IFI16, interferon-γ-inducible protein 16; IFN, interferon; LPS, lipopolysaccharide; MoMFs, monocyte-derived macrophages; NAFLD, non-alcoholic fatty liver disease; STING, stimulator of interferon genes; TBK1, TANK‐binding kinase 1; UC, ulcerative colitis; WT, wild-type.

## References

[B1] Ahn J. Gutman D. Saijo S. Barber G. N. (2012). STING manifests self DNA-dependent inflammatory disease. Proc. Natl. Acad. Sci. U. S. A. 109 (47), 19386–19391. 10.1073/pnas.1215006109 23132945 PMC3511090

[B2] Ahn J. Son S. Oliveira S. C. Barber G. N. (2017). STING-dependent signaling underlies IL-10 controlled inflammatory colitis. Cell Rep. 21 (13), 3873–3884. 10.1016/j.celrep.2017.11.101 29281834 PMC6082386

[B3] Ahn J. Xia T. Konno H. Konno K. Ruiz P. Barber G. N. (2014). Inflammation-driven carcinogenesis is mediated through STING. Nat. Commun. 5, 5166. 10.1038/ncomms6166 25300616 PMC4998973

[B4] Bai J. Liu F. (2019). The cGAS-cGAMP-STING pathway: A molecular link between immunity and metabolism. Diabetes 68 (6), 1099–1108. 10.2337/dbi18-0052 31109939 PMC6610018

[B5] Barber G. N. (2015). Sting: Infection, inflammation and cancer. Nat. Rev. Immunol. 15 (12), 760–770. 10.1038/nri3921 26603901 PMC5004891

[B6] Burdette D. L. Vance R. E. (2013). STING and the innate immune response to nucleic acids in the cytosol. Nat. Immunol. 14 (1), 19–26. 10.1038/ni.2491 23238760

[B7] Canesso M. C. C. Lemos L. Neves T. C. Marim F. M. Castro T. B. R. Veloso É S. (2018). The cytosolic sensor STING is required for intestinal homeostasis and control of inflammation. Mucosal Immunol. 11 (3), 820–834. 10.1038/mi.2017.88 29346345

[B8] Chaudhary G. Mahajan U. B. Goyal S. N. Ojha S. Patil C. R. Subramanya S. B. (2017). Protective effect of Lagerstroemia speciosa against dextran sulfate sodium induced ulcerative colitis in C57BL/6 mice. Am. J. Transl. Res. 9 (4), 1792–1800. 28469784 PMC5411927

[B9] Cheng Y. Hall T. R. Xu X. Yung I. Souza D. Zheng J. (2022). Targeting uPA-uPAR interaction to improve intestinal epithelial barrier integrity in inflammatory bowel disease. EBioMedicine 75, 103758. 10.1016/j.ebiom.2021.103758 34933179 PMC8688562

[B10] D'Haens G. Sandborn W. J. Feagan B. G. Geboes K. Hanauer S. B. Irvine E. J. (2007). A review of activity indices and efficacy end points for clinical trials of medical therapy in adults with ulcerative colitis. Gastroenterology 132 (2), 763–786. 10.1053/j.gastro.2006.12.038 17258735

[B11] Dieleman L. A. Ridwan B. U. Tennyson G. S. Beagley K. W. Bucy R. P. Elson C. O. (1994). Dextran sulfate sodium-induced colitis occurs in severe combined immunodeficient mice. Gastroenterology 107 (6), 1643–1652. 10.1016/0016-5085(94)90803-6 7958674

[B12] Ding R. Li H. Liu Y. Ou W. Zhang X. Chai H. (2022). Activating cGAS-STING axis contributes to neuroinflammation in CVST mouse model and induces inflammasome activation and microglia pyroptosis. J. Neuroinflammation 19 (1), 137. 10.1186/s12974-022-02511-0 35689216 PMC9188164

[B13] Ding Z. Mathur V. Ho P. P. James M. L. Lucin K. M. Hoehne A. (2014). Antiviral drug ganciclovir is a potent inhibitor of microglial proliferation and neuroinflammation. J. Exp. Med. 211 (2), 189–198. 10.1084/jem.20120696 24493798 PMC3920559

[B14] Donnelly C. R. Jiang C. Andriessen A. S. Wang K. Wang Z. Ding H. (2021). STING controls nociception via type I interferon signalling in sensory neurons. Nature 591 (7849), 275–280. 10.1038/s41586-020-03151-1 33442058 PMC7977781

[B15] Ekbom A. Helmick C. Zack M. Adami H. O. (1991). The epidemiology of inflammatory bowel disease: A large, population-based study in Sweden. Gastroenterology 100 (2), 350–358. 10.1016/0016-5085(91)90202-v 1985033

[B16] Elion G. B. Furman P. A. Fyfe J. A. de Miranda P. Beauchamp L. Schaeffer H. J. (1977). Selectivity of action of an antiherpetic agent, 9-(2-hydroxyethoxymethyl) guanine. Proc. Natl. Acad. Sci. U. S. A. 74 (12), 5716–5720. 10.1073/pnas.74.12.5716 202961 PMC431864

[B17] Elson C. O. Sartor R. B. Tennyson G. S. Riddell R. H. (1995). Experimental models of inflammatory bowel disease. Gastroenterology 109 (4), 1344–1367. 10.1016/0016-5085(95)90599-5 7557106

[B18] Faulds D. Heel R. C. (1990). Ganciclovir. A review of its antiviral activity, pharmacokinetic properties and therapeutic efficacy in cytomegalovirus infections. Drugs 39 (4), 597–638. 10.2165/00003495-199039040-00008 2161731

[B19] Fiske J. Liu E. Limdi J. K. Conley T. E. Townsend T. Davies M. (2022). Safety and effectiveness of ustekinumab in elderly Crohn's disease patients. Eur. J. Gastroenterol. Hepatol. 34 (11), 1132–1139. 10.1097/meg.0000000000002436 36170682

[B20] Graham D. B. Xavier R. J. (2020). Pathway paradigms revealed from the genetics of inflammatory bowel disease. Nature 578 (7796), 527–539. 10.1038/s41586-020-2025-2 32103191 PMC7871366

[B21] Green M. R. Sambrook J. (2018). Quantification of RNA by real-time reverse transcription-polymerase chain reaction (RT-PCR). Cold Spring Harb. Protoc. 2018 (10), pdb.prot095042. 10.1101/pdb.prot095042 30275077

[B22] Heipertz E. L. Harper J. Walker W. E. (2017). STING and TRIF contribute to mouse sepsis, depending on severity of the disease model. Shock 47 (5), 621–631. 10.1097/shk.0000000000000771 27755506

[B23] Hu Q. Ren H. Li G. Wang D. Zhou Q. Wu J. (2019). STING-mediated intestinal barrier dysfunction contributes to lethal sepsis. EBioMedicine 41, 497–508. 10.1016/j.ebiom.2019.02.055 30878597 PMC6443583

[B24] Huang H. Wang X. Zhang X. Zhang G. Jinbo M. Wang H. (2020). Ganciclovir reduces irinotecan-induced intestinal toxicity by inhibiting NLRP3 activation. Cancer Chemother. Pharmacol. 85 (1), 195–204. 10.1007/s00280-019-03996-y 31813002

[B25] Ji J. Shu D. Zheng M. Wang J. Luo C. Wang Y. (2016). Microbial metabolite butyrate facilitates M2 macrophage polarization and function. Sci. Rep. 6, 24838. 10.1038/srep24838 27094081 PMC4837405

[B26] Jostins L. Ripke S. Weersma R. K. Duerr R. H. McGovern D. P. Hui K. Y. (2012). Host-microbe interactions have shaped the genetic architecture of inflammatory bowel disease. Nature 491 (7422), 119–124. 10.1038/nature11582 23128233 PMC3491803

[B27] Ke X. Hu T. Jiang M. (2022). cGAS-STING signaling pathway in gastrointestinal inflammatory disease and cancers. Faseb J. 36 (1), e22029. 10.1096/fj.202101199R 34907606

[B28] Kilkenny C. Browne W. Cuthill I. C. Emerson M. Altman D. G. (2010). Animal research: Reporting *in vivo* experiments: The ARRIVE guidelines. Br. J. Pharmacol. 160 (7), 1577–1579. 10.1111/j.1476-5381.2010.00872.x 20649561 PMC2936830

[B29] Ko J. H. Peck K. R. Lee W. J. Lee J. Y. Cho S. Y. Ha Y. E. (2015). Clinical presentation and risk factors for cytomegalovirus colitis in immunocompetent adult patients. Clin. Infect. Dis. 60 (6), e20–e26. 10.1093/cid/ciu969 25452594

[B30] Kornbluth A. Sachar D. B. (2004). Ulcerative colitis practice guidelines in adults (update): American College of gastroenterology, practice parameters committee. Am. J. Gastroenterol. 99 (7), 1371–1385. 10.1111/j.1572-0241.2004.40036.x 15233681

[B31] Langholz E. Munkholm P. Davidsen M. Binder V. (1994). Course of ulcerative colitis: Analysis of changes in disease activity over years. Gastroenterology 107 (1), 3–11. 10.1016/0016-5085(94)90054-x 8020674

[B32] Langholz E. Munkholm P. Nielsen O. H. Kreiner S. Binder V. (1991). Incidence and prevalence of ulcerative colitis in Copenhagen county from 1962 to 1987. Scand. J. Gastroenterol. 26 (12), 1247–1256. 10.3109/00365529108998621 1763295

[B33] Lerang F. Holst R. Henriksen M. Wåhlberg H. Jelsness-Jørgensen L. P. (2022). Antitumour necrosis factor alpha treatment in Crohn's disease: Long-term efficacy, side effects and need for surgery. Scand. J. Gastroenterol. 57 (8), 921–929. 10.1080/00365521.2022.2042592 35188443

[B34] Liu C. Mo L. H. Feng B. S. Jin Q. R. Li Y. Lin J. (2021). Twist1 contributes to developing and sustaining corticosteroid resistance in ulcerative colitis. Theranostics 11 (16), 7797–7812. 10.7150/thno.62256 34335965 PMC8315068

[B35] Liu Y. Jesus A. A. Marrero B. Yang D. Ramsey S. E. Sanchez G. A. M. (2014). Activated STING in a vascular and pulmonary syndrome. N. Engl. J. Med. 371 (6), 507–518. 10.1056/NEJMoa1312625 25029335 PMC4174543

[B36] Luo X. Li H. Ma L. Zhou J. Guo X. Woo S. L. (2018). Expression of STING is increased in liver tissues from patients with NAFLD and promotes macrophage-mediated hepatic inflammation and fibrosis in mice. Gastroenterology 155 (6), 1971–1984. 10.1053/j.gastro.2018.09.010 30213555 PMC6279491

[B37] Martin G. R. Blomquist C. M. Henare K. L. Jirik F. R. (2019). Stimulator of interferon genes (STING) activation exacerbates experimental colitis in mice. Sci. Rep. 9 (1), 14281. 10.1038/s41598-019-50656-5 31582793 PMC6776661

[B38] Mathur V. Burai R. Vest R. T. Bonanno L. N. Lehallier B. Zardeneta M. E. (2017). Activation of the STING-dependent type I interferon response reduces microglial reactivity and neuroinflammation. Neuron 96 (6), 1290–1302. 10.1016/j.neuron.2017.11.032 29268096 PMC5806703

[B39] Matthews T. Boehme R. (1988). Antiviral activity and mechanism of action of ganciclovir. Rev. Infect. Dis. 10 (3), S490–S494. 10.1093/clinids/10.supplement_3.s490 2847285

[B40] Miao L. Qi J. Zhao Q. Wu Q. N. Wei D. L. Wei X. L. (2020). Targeting the STING pathway in tumor-associated macrophages regulates innate immune sensing of gastric cancer cells. Theranostics 10 (2), 498–515. 10.7150/thno.37745 31903134 PMC6929973

[B41] Nossa C. W. Oberdorf W. E. Yang L. Aas J. A. Paster B. J. Desantis T. Z. (2010). Design of 16S rRNA gene primers for 454 pyrosequencing of the human foregut microbiome. World J. Gastroenterol. 16 (33), 4135–4144. 10.3748/wjg.v16.i33.4135 20806429 PMC2932916

[B42] Ordás I. Eckmann L. Talamini M. Baumgart D. C. Sandborn W. J. (2012). Ulcerative colitis. Lancet 380 (9853), 1606–1619. 10.1016/s0140-6736(12)60150-0 22914296

[B43] Petrasek J. Iracheta-Vellve A. Csak T. Satishchandran A. Kodys K. Kurt-Jones E. A. (2013). STING-IRF3 pathway links endoplasmic reticulum stress with hepatocyte apoptosis in early alcoholic liver disease. Proc. Natl. Acad. Sci. U. S. A. 110 (41), 16544–16549. 10.1073/pnas.1308331110 24052526 PMC3799324

[B44] Pillet S. Pozzetto B. Roblin X. (2016). Cytomegalovirus and ulcerative colitis: Place of antiviral therapy. World J. Gastroenterol. 22 (6), 2030–2045. 10.3748/wjg.v22.i6.2030 26877608 PMC4726676

[B45] Poltorak A. He X. Smirnova I. Liu M. Y. Van Huffel C. Du X. (1998). Defective LPS signaling in C3H/HeJ and C57bl/10ScCr mice: Mutations in Tlr4 gene. Science 282 (5396), 2085–2088. 10.1126/science.282.5396.2085 9851930

[B46] Rugtveit J. Brandtzaeg P. Halstensen T. S. Fausa O. Scott H. (1994). Increased macrophage subset in inflammatory bowel disease: Apparent recruitment from peripheral blood monocytes. Gut 35 (5), 669–674. 10.1136/gut.35.5.669 8200563 PMC1374754

[B47] Schenk M. Bouchon A. Seibold F. Mueller C. (2007). TREM-1--expressing intestinal macrophages crucially amplify chronic inflammation in experimental colitis and inflammatory bowel diseases. J. Clin. Invest. 117 (10), 3097–3106. 10.1172/jci30602 17853946 PMC1974863

[B48] Schoggins J. W. MacDuff D. A. Imanaka N. Gainey M. D. Shrestha B. Eitson J. L. (2014). Pan-viral specificity of IFN-induced genes reveals new roles for cGAS in innate immunity. Nature 505 (7485), 691–695. 10.1038/nature12862 24284630 PMC4077721

[B49] Scott J. C. Partovi N. Ensom M. H. (2004). Ganciclovir in solid organ transplant recipients: Is there a role for clinical pharmacokinetic monitoring? Ther. Drug Monit. 26 (1), 68–77. 10.1097/00007691-200402000-00014 14749553

[B50] Shmuel-Galia L. Humphries F. Lei X. Ceglia S. Wilson R. Jiang Z. (2021). Dysbiosis exacerbates colitis by promoting ubiquitination and accumulation of the innate immune adaptor STING in myeloid cells. Immunity 54 (6), 1137–1153.e8. 10.1016/j.immuni.2021.05.008 34051146 PMC8237382

[B51] Skripuletz T. Salinas Tejedor L. Prajeeth C. K. Hansmann F. Chhatbar C. Kucman V. (2015). The antiviral drug ganciclovir does not inhibit microglial proliferation and activation. Sci. Rep. 5, 14935. 10.1038/srep14935 26447351 PMC4597339

[B52] Song J. H. Hong S. N. Kim E. R. Chang D. K. Kim Y. H. (2022). Performance of Remsima® monitor drug level versus ridascreen ifx monitoring in therapeutic drug monitoring of infliximab in patients with inflammatory bowel disease: A study of diagnostic accuracy. Med. Baltim. 101 (38), e30683. 10.1097/md.0000000000030683 PMC9509095 36197194

[B53] Taylor S. C. Posch A. (2014). The design of a quantitative Western blot experiment. Biomed. Res. Int. 2014, 361590. 10.1155/2014/361590 24738055 PMC3971489

[B54] Vlantis K. Polykratis A. Welz P. S. van Loo G. Pasparakis M. Wullaert A. (2016). TLR-independent anti-inflammatory function of intestinal epithelial TRAF6 signalling prevents DSS-induced colitis in mice. Gut 65 (6), 935–943. 10.1136/gutjnl-2014-308323 25761602 PMC4893119

[B55] Wang C. Z. Yao H. Zhang C. F. Chen L. Wan J. Y. Huang W. H. (2018a). American ginseng microbial metabolites attenuate DSS-induced colitis and abdominal pain. Int. Immunopharmacol. 64, 246–251. 10.1016/j.intimp.2018.09.005 30212750

[B56] Wang X. Rao H. Zhao J. Wee A. Li X. Fei R. (2020). STING expression in monocyte-derived macrophages is associated with the progression of liver inflammation and fibrosis in patients with nonalcoholic fatty liver disease. Lab. Invest. 100 (4), 542–552. 10.1038/s41374-019-0342-6 31745210

[B57] Wang Y. de Vallière C. Imenez Silva P. H. Leonardi I. Gruber S. Gerstgrasser A. (2018b). The proton-activated receptor GPR4 modulates intestinal inflammation. J. Crohns Colitis 12 (3), 355–368. 10.1093/ecco-jcc/jjx147 29136128

[B58] Wasserbauer M. Hlava S. Drabek J. Stovicek J. Minarikova P. Nedbalova L. (2022). Adalimumab biosimilars in the therapy of Crohn´s disease and ulcerative colitis: Prospective multicentric clinical monitoring. PLoS One 17 (8), e0271299. 10.1371/journal.pone.0271299 35939424 PMC9359532

[B59] Williams K. L. Fuller C. R. Dieleman L. A. DaCosta C. M. Haldeman K. M. Sartor R. B. (2001). Enhanced survival and mucosal repair after dextran sodium sulfate-induced colitis in transgenic mice that overexpress growth hormone. Gastroenterology 120 (4), 925–937. 10.1053/gast.2001.22470 11231946

[B60] Wirtz S. Neurath M. F. (2007). Mouse models of inflammatory bowel disease. Adv. Drug Deliv. Rev. 59 (11), 1073–1083. 10.1016/j.addr.2007.07.003 17825455

[B61] Wirtz S. Popp V. Kindermann M. Gerlach K. Weigmann B. Fichtner-Feigl S. (2017). Chemically induced mouse models of acute and chronic intestinal inflammation. Nat. Protoc. 12 (7), 1295–1309. 10.1038/nprot.2017.044 28569761

[B62] Xavier R. J. Podolsky D. K. (2007). Unravelling the pathogenesis of inflammatory bowel disease. Nature 448 (7152), 427–434. 10.1038/nature06005 17653185

[B63] Yan Y. X. Shao M. J. Qi Q. Xu Y. S. Yang X. Q. Zhu F. H. (2018). Artemisinin analogue SM934 ameliorates DSS-induced mouse ulcerative colitis via suppressing neutrophils and macrophages. Acta Pharmacol. Sin. 39 (10), 1633–1644. 10.1038/aps.2017.185 29849131 PMC6289314

[B64] Yao L. Y. Shao B. L. Tian F. Ye M. Li Y. Q. Wang X. L. (2022). Trends in medication use and treatment patterns in Chinese patients with inflammatory bowel disease. World J. Gastroenterol. 28 (30), 4102–4119. 10.3748/wjg.v28.i30.4102 36157116 PMC9403423

[B65] Zeng L. Kang R. Zhu S. Wang X. Cao L. Wang H. (2017). ALK is a therapeutic target for lethal sepsis. Sci. Transl. Med. 9 (412), eaan5689. 10.1126/scitranslmed.aan5689 29046432 PMC5737927

[B66] Zhang X. Bai X. C. Chen Z. J. (2020). Structures and mechanisms in the cGAS-STING innate immunity pathway. Immunity 53 (1), 43–53. 10.1016/j.immuni.2020.05.013 32668227

[B67] Zhao Q. Wei Y. Pandol S. J. Li L. Habtezion A. (2018). STING signaling promotes inflammation in experimental acute pancreatitis. Gastroenterology 154 (6), 1822–1835. 10.1053/j.gastro.2018.01.065 29425920 PMC6112120

[B68] Zhu Q. Man S. M. Gurung P. Liu Z. Vogel P. Lamkanfi M. (2014). Cutting edge: STING mediates protection against colorectal tumorigenesis by governing the magnitude of intestinal inflammation. J. Immunol. 193 (10), 4779–4782. 10.4049/jimmunol.1402051 25320273 PMC4308418

