# Supplementary material for: Low-dose ganciclovir ameliorates dextran sulfate sodium-induced ulcerative colitis through inhibiting macrophage STING activation in mice
Source: Front Pharmacol. 2022 Nov 17;13:1020670. doi: 10.3389/fphar.2022.1020670 (PMC9714675; doi:10.3389/fphar.2022.1020670)
Supplement: Supplementary file 1 [file DataSheet1.pdf]

Supplementary methods, Tables, and Figures

Materials and Methods

16S rRNA gene sequencing analysis

1. DNA extraction and amplification

On day 8 after 3% DSS treatment, All group mouse colon content samples were snap frozen and stored at −80 °C refrigerator after collection. Bacterial DNA was isolated from the colon contents using a MagPure Soil DNA LQ Kit (Magen, Guangdong, China) following the manufacturer's instructions. DNA concentration and integrity were measured by a NanoDrop 2000 spectrophotometer (Thermo Fisher Scientific, Waltham, MA, USA) and agarose gel electrophoresis, respectively. PCR amplification of the V3-V4 hypervariable regions of the bacterial 16S rRNA gene was carried out in a 25 μl reaction using universal primer pairs (343F :5'- TACGGRAGGCAGCAG -3'; 798R: 5'-AGGGTATCTAATCCT-3'). The reverse primer contained a sample barcode and both primers were connected with an Illumina sequencing adapter.

2. Library construction and sequencing

The Amplicon quality was visualized using gel electrophoresis. The PCR products were purified with Agencourt AMPure XP beads (Beckman Coulter Co., USA) and quantified using Qubit dsDNA assay kit. The concentrations were then adjusted for sequencing. Sequencing was performed on an Illumina NovaSeq6000 with two paired-end read cycles of 250 bases each.

3. Bioinformatic analysis

Paired-end reads were preprocessed using Trimmomatic software to detect and cut off ambiguous bases(N). It also cut off low quality sequences with average quality score below 20 using sliding window trimming approach. After trimming, paired-end reads were assembled using FLASH software. Parameters of assembly were: 10 bp of minimal overlapping, 200 bp of maximum overlapping and 20% of maximum mismatch rate. Sequences were performed further denoising as follows: reads with ambiguous, homologous sequences or below 200 bp were abandoned. Reads with 75% of bases above Q20 were retained using QIIME software (version 1.8.0). Then, reads with chimera were detected and removed using VSEARCH. Clean reads were subjected to primer sequences removal and clustering to generate operational taxonomic units (OTUs) using VSEARCH software with 97% similarity cutoff. The representative read of each OTU was selected using QIIME package. All representative reads were annotated and blasted against Silva database (Version 132) using RDP classifier (confidence threshold was 70%). The microbial diversity in colon content samples was estimated using the alpha diversity that include Chao1 index and Shannon index. The Unifrac distance matrix performed by QIIME software was used for unweighted Unifrac Principal coordinates analysis (PCoA). The 16S rRNA gene amplicon sequencing and analysis were cooperated by OE Biotech Co., Ltd. (Shanghai, China).

Supplementary Tables, Figures and legends

Supplementary Table 1: List of IBD studies used in meta-analysis

Project Name	Disease subtype	Biopsy Location	Disease samples	Control samples	All samples	Disease	Control	PMID
GSE16879	CD	colonic mucosa	CD before αTNF therapy	Control	13	19	6	19956723
GSE52746	CD	colon	Active CD without αTNF therapy	Healthy colonic samples from non-inflammatory controls	27	10	17	24700437
GSE59071	CD	colonic mucosa	CD active disease	Normal mucosa	19	8	11	26313692
GSE75214	CD	colonic mucosa	CD active disease	Control	19	8	11	28885228
GSE16879	UC	colonic mucosa	UC prior αTNF treatment	Control	30	24	6	19956723
GSE37283	UC	colonic mucosa	Non-quiescent UC	Normal controls	20	15	5	23945234
GSE59071	UC	colonic mucosa	UC active disease	Normal mucosa	108	97	11	26313692
GSE73661	UC	colonic mucosa	UC before treatment	Non IBD	43	35	8	27802155
GSE75214	UC	colonic mucosa	UC active disease	Control	85	74	11	28885228

Supplementary Table 2. Demographic data of human subjects CRP, C-reactive protein.

NLR: neutrophil-to-lymphocyte ratio.

Name	#1	# 2	# 3	# 4	# 5	# 6	#7	# 8
Age(year)	78	57	63	65	69	58	35	60
Gender	men	women	men	women	women	women	men	men
Disease duration	one month	four years	one year	one year	six years	five years	ten years	ten years
Clinical type	Colon cancer	Colon cancer	Colon cancer	Colon cancer	Ulcerative colitis	Ulcerative colitis	Ulcerative colitis	Ulcerative colitis
Disease location	carcinoma of the rectum	carcinoma of sigmoid	carcinoma of sigmoid	carcinoma of sigmoid				
Disease location					sigmoid colon	the duodenum department	terminal ileum	ascending colon
partial Mayo score	6	7	7	8				
WBC(/μL; ×1000)	7.2	7	4.5	5	5.2	5.3	5.3	8.9
Hemoglobin(g/dL)	150	135	152	139	153	136	143	147
Platelet(×104/μL)	220	204	63	195	169	220	181	315
Albumin (g/dL)	42.5	40.2	38.8	44.5	38.3	48	50.1	42.7
CRP(mg/dL)	5.5	2.5	4.6	4.7	52.7	5.6	7.8	7.1
NLR	2.32	2.42	0.74	2.75	2.33	1.45	2	2.39

Supplementary Table 3: KEY RESOURCES TABLE

REAGENT or	SOURCE	IDENTIFIER	Dilution
Antibodies			
Rabbit anti-STING	Novus, Colorado,USA	Cat#NBP2-24683	1:1000 (WB)<br>1:200 (IF)<br>1:300 (IHC)
rabbit anti-cGAS	Cell Signaling Technology, Boston, MA, USA	Cat#15102	1:2000 (WB)
rabbit anti-p-TBK1	Cell Signaling Technology, Boston, MA,USA	Cat# 5483	1:1000 (WB)
rabbit anti-TBK1	Abcam, Cambridgeshire, UK	Cat#ab40676	1:1000 (WB)
rabbit anti-IFN-β	Abcam, Cambridgeshire, UK	Cat#ab65783	1:1000 (WB)
rabbit anti- TNF-α	Abcam, Cambridgeshire, UK	Cat#ab205587	1:1000 (WB)
Mouse anti- IL-1β	Cell Signaling Technology, Boston, MA,USA	Cat#12242	1:2000 (WB)
rabbit anti-β-actin	ImmunoWay,Texas,USA	Cat#YT0099	1:2000 (WB)

Mouse anti-α-Tubulin	Affinity Biosciences,OH,USA	Cat#T0023	1:2000 (WB)
goat anti-mouse IgG secondary antibody	Thermo Fisher Scientific,Waltham, MA, USA	Cat# G-21040	1:2000 (WB)
goat anti-rabbit IgG secondary antibody	Thermo Fisher Scientific, Waltham, MA, USA	Cat#G-21234	1:2000 (WB)
anti F4/80 antibody	Abcam, Cambridgeshire, UK	Cat#ab6640	1:200 (IF)
goat anti-rabbit Alex Flour 555 pAb	Cell Signaling Technology, Boston, MA,USA	Cat#4413	1:200 (IF)
goat anti-rabbit Alex Flour 488 pAb	Abcam, Cambridgeshire, UK	Cat#ab150157	1:200 (IF)
Chemicals, Peptides and Recombinant Proteins			
Dextran Sulfate Sodium [illegible]	MP Biomedicals, Santa Ana, CA	Cat#160110	3mg/100ml
cGMP-AMP	Tocris Bioscience ,Bristol, UK	Cat#531889	10μM
Lipopolysaccharide	Sigma-Aldrich, St. Louis, MO, [illegible]	Cat#L2880	100ng/ml
DMXAA	Sigma-Aldrich, St. Louis, MO, [illegible]	Cat#D5817	20μg/ml
CMA	Shanghai yuanye Bio-Technology CO.,Ltd (Shanghai	Cat#S46701	250μg/ml
ganciclovir	China keyi Pharmaceutical CO.,Ltd (Wuhan City, Hubei Province, China)	Cat#H20030419	10mg/ml<br>50μM
Fecal occult blood test, F0BT	Shanghai bailai Bio-Technology CO.,Ltd(Shanghai City, China)	Cat#FD9349	

Supplementary Table 4: Listed statistical analyses used within the paper.

Figures	Test used	Data structure	Comparison	F value or t value	*P* value
Fig.1A	one-way ANOVA	Normally distributed	cGAS for different concentration of GCV vs. saline.	$F_{(7,24)}$=5.490	*P* = 0.0007;
Fig.1B	one-way ANOVA	Normally distributed	STING for different concentration of GCV vs. saline.	$F_{(7,24)}$= 18.80	*P* <0.0001 ;
Fig.1C	one-way ANOVA	Normally distributed	IL-10 for different concentration of GCV vs. saline.	$F_{(7,24)}$= 22.32	*P* <0.0001 ;
Fig.1D	one-way	Normally	IFN-β for different	$F_{(7,24)}$= 65.25	*P*

	ANOVA	distributed	concentration of GCV vs. saline.		<0.0001 ;
Fig.1E	one-way ANOVA	Normally distributed	CXCL-10 for different concentration of GCV vs. saline.	$F_{(7,24)}$= 7.336	P < 0.0001 ;
Fig.1F	Log-rank test	Not Normally distributed	10 mg/kg GCV plus LPS vs. LPS plus saline.	Chi square=4.870 df=4	P = 0.0273;
Fig.1F	Log-rank test	Not Normally distributed	100 mg/kg GCV plus LPS vs. LPS plus saline.	Chi square=3.882 df=4	P = 0.0488;
Fig.1H	unpaired Student's *t*-test	Normally distributed	100 ng LPS vs. saline.	t= 7.433 df=5	P = 0.0007
Fig.1H	unpaired Student's *t*-test	Normally distributed	50 µM GCV plus 100 ng LPS vs. 100 ng LPS	t = 4.704 df= 5	P = 0.0053
Fig.1H	unpaired Student's *t*-test	Normally distributed	100 ng LPS vs. saline.	t = 4.017 df= 4	P = 0.0159
Fig.1H	unpaired Student's *t*-test	Normally distributed	50 µM GCV plus 100 ng LPS vs. 100 ng LPS	t = 4.694 df= 4	P = 0.0093
Fig. 1I	unpaired Student's *t*-test	Normally distributed	100 ng LPS vs. saline.	t = 5.328 df=6	P = 0.0018
Fig. 1I	unpaired Student's *t*-test	Normally distributed	50 µM GCV plus 100 ng LPS vs. 100 ng LPS	t = 6.579 df= 6	P = 0.0006
Fig. 1I	unpaired Student's *t*-test	Normally distributed	100 ng LPS vs. saline.	t = 14.15 df=6	P < 0.0001
Fig.1I	unpaired Student's *t*-test	Normally distributed	50 µM GCV plus 100 ng LPS vs. 100 ng LPS	t= 9.378 df= 6	P < 0.0001
Fig.1I	unpaired Student's *t*-test	Normally distributed	100 ng LPS vs. saline.	t= 6.001 df=6	P = 0.0010
Fig.1I	unpaired Student's *t*-test	Normally distributed	50 µM GCV plus 100 ng LPS vs. 100 ng LPS	t = 5.147 df=6	P = 0.0021
Fig.2A	unpaired Student's *t*-test	Normally distributed	STING: 250 µg/ml CMA vs. saline.	t= 12.79 df = 6	P < 0.0001;
Fig.2A	unpaired Student's *t*-test	Normally distributed	STING: 50 µM GCV plus 250 µg/ml CMA vs. 250 µg/ml CMA.	t= 13.53 df= 6	P < 0.0001;
Fig.2A	unpaired	Normally	IL-10:	t = 14.49	P <

	Student's *t*-test	distributed	250 µg/ml CMA vs. saline.	df= 6	0.0001;
Fig.2A	unpaired Student's *t*-test	Normally distributed	IL-10: 50 µM GCV plus 250 µg/ml CMA vs. 250 µg/ml CMA.	t= 12.83 df = 6	*P* <0.0001;
Fig.2A	unpaired Student's *t*-test	Normally distributed	IFN-β: 250 µg/ml CMA vs. saline.	t = 4.792 df=6	*P* = 0.0030;
Fig.2A	unpaired Student's *t*-test	Normally distributed	IFN-β: 50 µM GCV plus 250 µg/ml CMA vs. 250 µg/ml CMA.	t = 6.620 df= 6	*P* = 0.0006;
Fig.2B	unpaired Student's *t*-test	Normally distributed	STING: 20 µg/ml DMXAA vs. saline.	t =3.046 df= 6	*P* = 0.226;
Fig.2B	unpaired Student's *t*-test	Normally distributed	STING: 50 µM GCV plus 20 µg/ml DMXAA vs. 20 µg/ml DMXAA.	t = 4.711 df=6	*P* = 0.0033;
Fig.2B	unpaired Student's *t*-test	Normally distributed	IL-10: 20 µg/ml DMXAA vs. saline.	t = 5.473 df= 6	*P* = 0.0016;
Fig.2B	unpaired Student's *t*-test	Normally distributed	IL-10: 50 µM.GCV plus 20 µg/ml DMXAA vs. 20 µg/ml DMXAA	t= 3.844 df=6	*P* = 0.0085;
Fig.2B	unpaired Student's *t*-test	Normally distributed	IFN-β: 20 µg/ml DMXAA vs. saline.	t=26.99 df=6	*P* < 0.0001
Fig.2B	unpaired Student's *t*-test	Normally distributed	IFN-β: 50 µM.GCV plus 20 µg/ml DMXAA vs. 20 µg/ml DMXAA	t= 25.86 df= 6	*P* < 0.0001
Fig.2C	unpaired Student's *t*-test	Normally distributed	STING: 10 µM cGAMP vs. saline.	t = 10.04 df= 6	*P* < 0.0001 ;
Fig. 2C	unpaired Student's *t*-test	Normally distributed	STING: 50 µM GCV plus 10 µM cGAMP vs. 10 µM cGAMP	t = 4.972 df=6	*P* = 0.0025;
Fig.2C	unpaired Student's *t*-test	Normally distributed	IL-10: 10 µM cGAMP vs. saline.	t = 21.83 df= 6	*P* <0.0001 ;
Fig.2C	unpaired Student's *t*-test	Normally distributed	IL-10: 50 µM GCV plus 10 µM cGAMP vs.10 µM cGAMP	t = 16.30 df= 6	*P* < 0.0001 ;
Fig. 2C	unpaired Student's *t*-	Normally distributed	IFN-β: 10 µM cGAMP vs.	t = 9.755 df= 6	*P* < 0.0001;

	test		saline.		
Fig. 2C	unpaired Student’s *t*-test	Normally distributed	IFN-β: 50 μM GCV plus 10 μM cGAMP vs. 10 μM cGAMP	t = 8.662 df= 6	*P* = 0.0001;
Fig.2E	unpaired Student’s *t*-test	Normally distributed	cGAS: 250 μg/ml CMA vs. saline.	t = 3.482 df= 4	*P* = 0.0253;
Fig.2E	unpaired Student’s *t*-test	Normally distributed	cGAS: 50 μM GCV plus 250 μg/ml CMA vs. 250 μg/ml CMA.	t= 4.916 df= 4	*P* = 0.0080 ;
Fig.2E	unpaired Student’s *t*-test	Normally distributed	STING: 250 μg/ml CMA vs. saline.	t= 5.141 df= 5	*P* =0.0036 ;
Fig.2E	unpaired Student’s *t*-test	Normally distributed	STING: 50 μM GCV plus 250 μg/ml CMA vs. 250 μg/ml CMA.	t= 6.002 df= 5	*P* = 0.0018 ;
Fig.2E	unpaired Student’s *t*-test	Normally distributed	IFN-β: 250 μg/ml CMA vs. saline.	t = 3.399 df= 4	*P* = 0.0273;
Fig.2E	unpaired Student’s *t*-test	Normally distributed	IFN-β: 50 μM GCV plus 250 μg/ml CMA vs. 250 μg/ml CMA.	t= 3.563 df= 4	*P* = 0.0235;
Fig.2E	unpaired Student’s *t*-test	Normally distributed	p-TBK1: 250 μg/ml CMA vs. saline.	t = 4.325 df=5	*P* = 0.0075;
Fig.2E	unpaired Student’s *t*-test	Normally distributed	p-TBK1: 50 μM GCV plus 250 μg/ml CMA vs. 250 μg/ml CMA.	t= 4.821 df= 5	*P* = 0.0048;
Fig.2G	unpaired Student’s *t*-test	Normally distributed	cGAS: 20 μg/ml DMXAA vs. saline.	t = 4.220 df= 4	*P* = 0.0135;
Fig.2G	unpaired Student’s *t*-test	Normally distributed	cGAS: 50 μM GCV plus 20 μg/ml DMXAA vs. 20 μg/ml DMXAA.	t= 2.919 df= 4	*P* = 0.0433 ;
Fig.2G	unpaired Student’s *t*-test	Normally distributed	STING: 20 μg/ml DMXAA vs. saline.	t= 0.0217 df=4	*P* = 0.0217;
Fig.2G	unpaired Student’s *t*-test	Normally distributed	STING: 50 μM GCV plus 20 μg/ml DMXAA vs. 20 μg/ml DMXAA.	t= 3.197 df= 4	*P* = 0.0330;
Fig.2G	unpaired Student’s *t*-test	Normally distributed	IFN-β: 20 μg/ml DMXAA vs. saline.	t = 3.283 df= 4	*P* = 0.0304 ;

Fig.2G	unpaired Student's *t*-test	Normally distributed	IFN-β: 50 µM GCV plus 20 µg/ml DMXAA vs. 20 µg/ml DMXAA.	t = 3.651 df= 5	*P* = 0.0147;
Fig.2G	unpaired Student's *t*-test	Normally distributed	p-TBK1: 20 µg/ml DMXAA vs. saline.	t = 6.049 df= 4	*P* = 0.0038;
Fig.2G	unpaired Student's *t*-test	Normally distributed	p-TBK1: 50 µM GCV plus 20 µg/ml DMXAA vs. 20 µg/ml DMXAA.	t= 3.058 df= 5	*P* = 0.0282;
Fig.2I	unpaired Student's *t*-test	Normally distributed	p-TBk1: 10 µM cGAMP vs. saline.	t= 5.717 df= 4	*P* = 0.0046;
Fig. 2I	unpaired Student's *t*-test	Normally distributed	p-TBK1: 50 µM GCV plus 10 µM cGAMP vs. 10 µM cGAMP	t= 6.279 df= 4	*P* = 0.0033;
Fig. 2I	unpaired Student's *t*-test	Normally distributed	cGAS: 10 µM cGAMP vs. saline.	t= 2.915 df= 6	*P* = 0.0268 ;
Fig. 2I	unpaired Student's *t*-test	Normally distributed	cGAS: 50 µM GCV plus 10 µM cGAMP vs. 10µM cGAMP	t= 4.242 df= 6	*P* = 0.0054;
Fig. 2I	unpaired Student's *t*-test	Normally distributed	STING: 10 µM cGAMP vs. saline.	t = 6.084 df= 5	*P* = 0.0017;
Fig. 2I	unpaired Student's *t*-test	Normally distributed	STING: 50 µM GCV plus 10 µM cGAMP vs. 10 µM cGAMP	t= 5.907 df= 6	*P* = 0.0010;
Fig. 2I	unpaired Student's *t*-test	Normally distributed	IFN-β: 10 µM cGAMP vs. saline.	t = 8.894 df=4	*P* = 0.0009 ;
Fig.2I	unpaired Student's *t*-test	Normally distributed	IFN-β: 50 µM GCV plus 10 µM cGAMP vs. 10 µM cGAMP	t= 3.584 df=4	*P* = 0.0231;
Fig.3A Macrophage UC GSE16879 CD68	unpaired Student's *t*-test	Normally distributed	Control VS Ulcerative Colitis	t=4.040, df=28	0.0004
Fig.3A Macrophage UC GSE37283 CD68	unpaired Student's *t*-test	Normally distributed	Control VS Crohn's Disease colitis	t=1.264, df=18	0.2222

Fig.3A Macrophage UC GSE59017 CD68	unpaired Student's *t*-test	Normally distributed	Control VS Ulcerative Colitis	t=3.351, df=106	0.0011
Fig.3A Macrophage UC GSE73661 CD68	unpaired Student's *t*-test	Normally distributed	Control VS Crohn's Disease colitis	t=5.135, df=41	<0.0001
Fig.3A Macrophage UC GSE75214 CD68	unpaired Student's *t*-test	Normally distributed	Control VS Crohn's Disease colitis	t=5.089, df=83	<0.0001
Fig.3A Macrophage CD GSE16879 CD68	unpaired Student's *t*-test	Normally distributed	Control VS Crohn's Disease colitis	t=3.495, df=23	0.0019
Fig.3A Macrophage CD GSE52746 CD68	unpaired Student's *t*-test	Normally distributed	Control VS Crohn's Disease colitis	t=1.719, df=25	0.0980
Fig.3A Macrophage CD GSE59071 CD68	unpaired Student's *t*-test	Normally distributed	Control VS Crohn's Disease colitis	t=3.499, df=17	0.0028
Fig.3A Macrophage CD GSE75214 CD68	unpaired Student's *t*-test	Normally distributed	Control VS Crohn's Disease colitis	t=3.541, df=17	0.0025
Fig.3A Macrophage UC GSE16879 CD80	unpaired Student's *t*-test	Normally distributed	Control VS Ulcerative Colitis	t=2.291, df=28	0.0297
Fig.3A Macrophage UC GSE37283 CD80	unpaired Student's *t*-test	Normally distributed	Control VS Crohn's Disease colitis	t=0.6916, df=18	0.4980
Fig.3A Macrophage UC GSE59017 CD80	unpaired Student's *t*-test	Normally distributed	Control VS Ulcerative Colitis	t=3.021, df=106	0.0032

Fig.3A Macrophage UC GSE73661 CD80	unpaired Student's *t*-test	Normally distributed	Control VS Crohn's Disease colitis	t=2.800, df=41	0.0078
Fig.3A Macrophage UC GSE75214 CD80	unpaired Student's *t*-test	Normally distributed	Control VS Crohn's Disease colitis	t=3.973, df=83	0.0002
Fig.3A Macrophage CD GSE16879 CD80	unpaired Student's *t*-test	Normally distributed	Control VS Crohn's Disease colitis	t=1.203, df=23	0.2412
Fig.3A Macrophage CD GSE52746 CD80	unpaired Student's *t*-test	Normally distributed	Control VS Crohn's Disease colitis	t=3.983, df=25	0.0005
Fig.3A Macrophage CD GSE59071 CD80	unpaired Student's *t*-test	Normally distributed	Control VS Crohn's Disease colitis	t=4.097, df=17	0.0008
Fig.3A Macrophage CD GSE75214 CD80	unpaired Student's *t*-test	Normally distributed	Control VS Crohn's Disease colitis	t=4.177, df=17	0.0006
Fig.3A Macrophage UC GSE16879 CD86	unpaired Student's *t*-test	Normally distributed	Control VS Ulcerative Colitis	t=3.458, df=28	0.0018
Fig.3A Macrophage UC GSE37283 CD86	unpaired Student's *t*-test	Normally distributed	Control VS Crohn's Disease colitis	t=2.899, df=18	0.0096
Fig.3A Macrophage UC GSE59017 CD86	unpaired Student's *t*-test	Normally distributed	Control VS Ulcerative Colitis	t=2.888, df=106	0.0047
Fig.3A Macrophage UC GSE73661 CD86	unpaired Student's *t*-test	Normally distributed	Control VS Crohn's Disease colitis	t=3.357, df=41	0.0017

Fig.3A Macrophage UC GSE75214 CD86	unpaired Student's *t*-test	Normally distributed	Control VS Crohn's Disease colitis	t=3.902, df=83	0.0002
Fig.3A Macrophage CD GSE16879 CD86	unpaired Student's *t*-test	Normally distributed	Control VS Crohn's Disease colitis	t=4.228, df=23	0.0003
Fig.3A Macrophage CD GSE52746 CD86	unpaired Student's *t*-test	Normally distributed	Control VS Crohn's Disease colitis	t=2.970, df=25	0.0065
Fig.3A Macrophage CD GSE59071 CD86	unpaired Student's *t*-test	Normally distributed	Control VS Crohn's Disease colitis	t=3.016, df=17	0.0078
Fig.3A Macrophage CD GSE75214 CD86	unpaired Student's *t*-test	Normally distributed	Control VS Crohn's Disease colitis	t=2.990, df=17	0.0082
Fig.3A Macrophage UC GSE16879 CD163	unpaired Student's *t*-test	Normally distributed	Control VS Ulcerative Colitis	t=1.495, df=28	0.1462
Fig.3A Macrophage UC GSE37283 CD163	unpaired Student's *t*-test	Normally distributed	Control VS Crohn's Disease colitis	t=3.199, df=18	0.0050
Fig.3A Macrophage UC GSE59017 CD163	unpaired Student's *t*-test	Normally distributed	Control VS Ulcerative Colitis	t=2.166, df=106	0.0326
Fig.3A Macrophage UC GSE73661 CD163	unpaired Student's *t*-test	Normally distributed	Control VS Crohn's Disease colitis	t=2.265, df=41	0.0288
Fig.3A Macrophage UC GSE75214 CD163	unpaired Student's *t*-test	Normally distributed	Control VS Crohn's Disease colitis	t=2.766, df=83	0.0070

Fig.3A Macrophage CD GSE16879 CD163	unpaired Student's *t*-test	Normally distributed	Control VS Crohn's Disease colitis	t=1.881, df=23	0.0727
Fig.3A Macrophage CD GSE52746 CD163	unpaired Student's *t*-test	Normally distributed	Control VS Crohn's Disease colitis	t=2.399, df=25	0.0242
Fig.3A Macrophage CD GSE59071 CD163	unpaired Student's *t*-test	Normally distributed	Control VS Crohn's Disease colitis	t=2.810, df=17	0.0120
Fig.3A Macrophage CD GSE75214 CD163	unpaired Student's *t*-test	Normally distributed	Control VS Crohn's Disease colitis	t=2.706, df=17	0.0150
Fig.3A cGAS-STING UC GSE16879 Sting	unpaired Student's *t*-test	Normally distributed	Control VS Ulcerative Colitis	t=4.252, df=28	0.0002
Fig.3A cGAS-STING UC GSE37283 Sting	unpaired Student's *t*-test	Normally distributed	Control VS Crohn's Disease colitis	t=0.4938, df=18	0.6274
Fig.3A cGAS-STING UC GSE59017 Sting	unpaired Student's *t*-test	Normally distributed	Control VS Ulcerative Colitis	t=5.782, df=106	<0.0001
Fig.3A cGAS-STING UC GSE73661 Sting	unpaired Student's *t*-test	Normally distributed	Control VS Crohn's Disease colitis	t=6.461, df=41	<0.0001
Fig.3A cGAS-STING UC GSE75214 Sting	unpaired Student's *t*-test	Normally distributed	Control VS Crohn's Disease colitis	t=5.493, df=83	<0.0001
Fig.3A cGAS-STING CD GSE16879 Sting	unpaired Student's *t*-test	Normally distributed	Control VS Crohn's Disease colitis	t=4.742, df=23	<0.0001

Fig.3A cGAS-STING CD GSE52746 Sting	unpaired Student's *t*-test	Normally distributed	Control VS Crohn's Disease colitis	t=4.415, df=25	0.0002
Fig.3A cGAS-STING CD GSE59071 Sting	unpaired Student's *t*-test	Normally distributed	Control VS Crohn's Disease colitis	t=6.427, df=17	<0.0001
Fig.3A cGAS-STING CD GSE75214 CD163	unpaired Student's *t*-test	Normally distributed	Control VS Crohn's Disease colitis	t=5.436, df=17	<0.0001
Fig.3A cGAS-STING UC GSE16879 cGAS	unpaired Student's *t*-test	Normally distributed	Control VS Ulcerative Colitis	t=3.552, df=28	0.0014
Fig.3A cGAS-STING UC GSE37283 cGAS	unpaired Student's *t*-test	Normally distributed	Control VS Crohn's Disease colitis	t=1.497, df=18	0.1516
Fig.3A cGAS-STING UC GSE59017 cGAS	unpaired Student's *t*-test	Normally distributed	Control VS Ulcerative Colitis	t=3.874, df=106	0.0002
Fig.3A cGAS-STING UC GSE73661 cGAS	unpaired Student's *t*-test	Normally distributed	Control VS Crohn's Disease colitis	t=4.793, df=41	<0.0001
Fig.3A cGAS-STING UC GSE75214 cGAS	unpaired Student's *t*-test	Normally distributed	Control VS Crohn's Disease colitis	t=5.461, df=83	<0.0001
Fig.3A cGAS-STING CD GSE16879 cGAS	unpaired Student's *t*-test	Normally distributed	Control VS Crohn's Disease colitis	t=4.537, df=23	0.0001
Fig.3A cGAS-STING CD GSE52746 cGAS	unpaired Student's *t*-test	Normally distributed	Control VS Crohn's Disease colitis	t=3.887, df=25	0.0007

Fig.3A cGAS-STING CD GSE59071 cGAS	unpaired Student's *t*-test	Normally distributed	Control VS Crohn's Disease colitis	t=3.221, df=17	0.0050
Fig.3A cGAS-STING CD GSE75214 cGAS	unpaired Student's *t*-test	Normally distributed	Control VS Crohn's Disease colitis	t=3.411, df=17	0.0033
Fig.3A Inflammatory UC GSE16879 IL-10	unpaired Student's *t*-test	Normally distributed	Control VS Ulcerative Colitis	t=2.855, df=28	0.0080
Fig.3A Inflammatory UC GSE37283 IL-10	unpaired Student's *t*-test	Normally distributed	Control VS Crohn's Disease colitis	t=1.210, df=18	0.2419
Fig.3A Inflammatory UC GSE59017 IL-10	unpaired Student's *t*-test	Normally distributed	Control VS Ulcerative Colitis	t=1.807, df=106	0.0736
Fig.3A Inflammatory UC GSE73661 IL-10	unpaired Student's *t*-test	Normally distributed	Control VS Crohn's Disease colitis	t=1.636, df=41	0.1095
Fig.3A Inflammatory UC GSE75214 IL-10	unpaired Student's *t*-test	Normally distributed	Control VS Crohn's Disease colitis	t=2.240, df=83	0.0278
Fig.3A Inflammatory CD GSE16879 IL-10	unpaired Student's *t*-test	Normally distributed	Control VS Crohn's Disease colitis	t=1.926, df=23	0.0665
Fig.3A Inflammatory CD GSE52746 IL-10	unpaired Student's *t*-test	Normally distributed	Control VS Crohn's Disease colitis	t=0.1931, df=25	0.8484
Fig.3A Inflammatory CD GSE59071 IL-10	unpaired Student's *t*-test	Normally distributed	Control VS Crohn's Disease colitis	t=1.544, df=17	0.1411

Fig.3A Inflammatory CD GSE75214 IL-10	unpaired Student's *t*-test	Normally distributed	Control VS Crohn's Disease colitis	t=1.841, df=17	0.0831
Fig.3A Inflammatory UC GSE16879 IL-1B	unpaired Student's *t*-test	Normally distributed	Control VS Ulcerative Colitis	t=2.451, df=28	0.0208
Fig.3A Inflammatory UC GSE37283 IL-1B	unpaired Student's *t*-test	Normally distributed	Control VS Crohn's Disease colitis	t=1.652, df=18	0.1158
Fig.3A Inflammatory UC GSE59017 IL-1B	unpaired Student's *t*-test	Normally distributed	Control VS Ulcerative Colitis	t=2.492, df=106	0.0143
Fig.3A Inflammatory UC GSE73661 IL-1B	unpaired Student's *t*-test	Normally distributed	Control VS Crohn's Disease colitis	t=2.488, df=41	0.0170
Fig.3A Inflammatory UC GSE75214 IL-1B	unpaired Student's *t*-test	Normally distributed	Control VS Crohn's Disease colitis	t=2.954, df=83	0.0041
Fig.3A Inflammatory CD GSE16879 IL-1B	unpaired Student's *t*-test	Normally distributed	Control VS Crohn's Disease colitis	t=2.251, df=23	0.0342
Fig.3A Inflammatory CD GSE52746 IL-1B	unpaired Student's *t*-test	Normally distributed	Control VS Crohn's Disease colitis	t=3.346, df=25	0.0026
Fig.3A Inflammatory CD GSE59071 IL-1B	unpaired Student's *t*-test	Normally distributed	Control VS Crohn's Disease colitis	t=2.388, df=17	0.0288
Fig.3A Inflammatory CD GSE75214 IL-1B	unpaired Student's *t*-test	Normally distributed	Control VS Crohn's Disease colitis	t=2.377, df=17	0.0294

Fig.3A Interferon 1 UC GSE16879 IFNA2	unpaired Student's *t*-test	Normally distributed	Control VS Ulcerative Colitis	t=1.028, df=28	0.3129
Fig.3A Interferon 1 UC GSE37283 IFNA2	unpaired Student's *t*-test	Normally distributed	Control VS Crohn's Disease colitis	t=2.740, df=18	0.0135
Fig.3A Interferon 1 UC GSE59017 IFNA2	unpaired Student's *t*-test	Normally distributed	Control VS Ulcerative Colitis	t=1.688, df=106	0.0943
Fig.3A Interferon 1 UC GSE73661 IFNA2	unpaired Student's *t*-test	Normally distributed	Control VS Crohn's Disease colitis	t=0.4278, df=41	0.6710
Fig.3A Interferon 1 UC GSE75214 IFNA2	unpaired Student's *t*-test	Normally distributed	Control VS Crohn's Disease colitis	t=2.905, df=83	0.0047
Fig.3A Interferon 1 CD GSE16879 IFNA2	unpaired Student's *t*-test	Normally distributed	Control VS Crohn's Disease colitis	t=0.02167, df=22	0.9829
Fig.3A Interferon 1 CD GSE52746 IFNA2	unpaired Student's *t*-test	Normally distributed	Control VS Crohn's Disease colitis	t=1.117, df=25	0.2745
Fig.3A Interferon 1 CD GSE59071 IFNA2	unpaired Student's *t*-test	Normally distributed	Control VS Crohn's Disease colitis	t=0.7734, df=17	0.4499
Fig.3A Interferon 1 CD GSE75214 IFNA2	unpaired Student's *t*-test	Normally distributed	Control VS Crohn's Disease colitis	t=0.9248, df=17	0.3680
Fig.3A Interferon 1 UC GSE16879 IFNB1	unpaired Student's *t*-test	Normally distributed	Control VS Ulcerative Colitis	t=0.3523, df=28	0.7272

Fig.3A Interferon 1 UC GSE37283 IFNB1	unpaired Student's *t*-test	Normally distributed	Control VS Crohn's Disease colitis	t=0.2609, df=18	0.7972
Fig.3A Interferon 1 UC GSE59017 IFNB1	unpaired Student's *t*-test	Normally distributed	Control VS Ulcerative Colitis	t=1.111, df=106	0.2689
Fig.3A Interferon 1 UC GSE73661 IFNB1	unpaired Student's *t*-test	Normally distributed	Control VS Crohn's Disease colitis	t=1.140, df=41	0.2610
Fig.3A Interferon 1 UC GSE75214 IFNB1	unpaired Student's *t*-test	Normally distributed	Control VS Crohn's Disease colitis	t=2.209, df=83	0.0299
Fig.3A Interferon 1 CD GSE16879 IFNB1	unpaired Student's *t*-test	Normally distributed	Control VS Crohn's Disease colitis	t=0.7935, df=23	0.4356
Fig.3A Interferon 1 CD GSE52746 IFNB1	unpaired Student's *t*-test	Normally distributed	Control VS Crohn's Disease colitis	t=0.4535, df=25	0.6541
Fig.3A Interferon 1 CD GSE59071 IFNB1	unpaired Student's *t*-test	Normally distributed	Control VS Crohn's Disease colitis	t=1.612, df=17	0.1255
Fig.3A Interferon 1 CD GSE75214 IFNB1	unpaired Student's *t*-test	Normally distributed	Control VS Crohn's Disease colitis	t=1.323, df=17	0.2034
Fig.3A Interferon 1 UC GSE16879 IFNAR1	unpaired Student's *t*-test	Normally distributed	Control VS Ulcerative Colitis	t=0.8958, df=28	0.3780
Fig.3A Interferon 1 UC GSE37283 IFNAR1	unpaired Student's *t*-test	Normally distributed	Control VS Crohn's Disease colitis	t=2.402, df=18	0.0273

Fig.3A Interferon 1 UC GSE59017 IFNAR1	unpaired Student's *t*-test	Normally distributed	Control VS Ulcerative Colitis	t=0.7884, df=106	0.4322
Fig.3A Interferon 1 UC GSE73661 IFNAR1	unpaired Student's *t*-test	Normally distributed	Control VS Crohn's Disease colitis	t=0.8743, df=41	0.3870
Fig.3A Interferon 1 UC GSE75214 IFNAR1	unpaired Student's *t*-test	Normally distributed	Control VS Crohn's Disease colitis	t=1.411, df=83	0.1620
Fig.3A Interferon 1 CD GSE16879 IFNAR1	unpaired Student's *t*-test	Normally distributed	Control VS Crohn's Disease colitis	t=0.9317, df=23	0.3612
Fig.3A Interferon 1 CD GSE52746 IFNAR1	unpaired Student's *t*-test	Normally distributed	Control VS Crohn's Disease colitis	t=1.445, df=25	0.1609
Fig.3A Interferon 1 CD GSE59071 IFNAR1	unpaired Student's *t*-test	Normally distributed	Control VS Crohn's Disease colitis	t=0.6388, df=17	0.5315
Fig.3A Interferon 1 CD GSE75214 IFNAR1	unpaired Student's *t*-test	Normally distributed	Control VS Crohn's Disease colitis	t=0.7830, df=17	0.4444
Fig.3A Interferon 1 UC GSE16879 IFNAR2	unpaired Student's *t*-test	Normally distributed	Control VS Ulcerative Colitis	t=3.267, df=28	0.0029
Fig.3A Interferon 1 UC GSE37283 IFNAR2	unpaired Student's *t*-test	Normally distributed	Control VS Crohn's Disease colitis	t=3.342, df=18	0.0036
Fig.3A Interferon 1 UC GSE59017 IFNAR2	unpaired Student's *t*-test	Normally distributed	Control VS Ulcerative Colitis	t=3.781, df=106	0.0003

Fig.3A Interferon 1 UC GSE73661 IFNAR2	unpaired Student's *t*-test	Normally distributed	Control VS Crohn's Disease colitis	t=5.207, df=41	<0.0001
Fig.3A Interferon 1 UC GSE75214 IFNAR2	unpaired Student's *t*-test	Normally distributed	Control VS Crohn's Disease colitis	t=5.995, df=83	<0.0001
Fig.3A Interferon 1 CD GSE16879 IFNAR2	unpaired Student's *t*-test	Normally distributed	Control VS Crohn's Disease colitis	t=2.249, df=23	0.0344
Fig.3A Interferon 1 CD GSE52746 IFNAR2	unpaired Student's *t*-test	Normally distributed	Control VS Crohn's Disease colitis	t=2.522, df=25	0.0184
Fig.3A Interferon 1 CD GSE59071 IFNAR2	unpaired Student's *t*-test	Normally distributed	Control VS Crohn's Disease colitis	t=2.639, df=17	0.0172
Fig.3A Interferon 1 CD GSE75214 IFNAR2	unpaired Student's *t*-test	Normally distributed	Control VS Crohn's Disease colitis	t=2.626, df=17	0.0177
Fig.3A Interferon 2 UC GSE16879 IFG	unpaired Student's *t*-test	Normally distributed	Control VS Ulcerative Colitis	t=2.303, df=28	0.0289
Fig.3A Interferon 2 UC GSE37283 IFG	unpaired Student's *t*-test	Normally distributed	Control VS Crohn's Disease colitis	t=0.2663, df=18	0.7930
Fig.3A Interferon 2 UC GSE59017 IFG	unpaired Student's *t*-test	Normally distributed	Control VS Ulcerative Colitis	t=1.540, df=106	0.1265
Fig.3A Interferon 2 UC GSE73661 IFG	unpaired Student's *t*-test	Normally distributed	Control VS Crohn's Disease colitis	t=2.022, df=41	0.0497

Fig.3A Interferon 2 UC GSE75214 IFG	unpaired Student's *t*-test	Normally distributed	Control VS Crohn's Disease colitis	t=1.838, df=83	0.0696
Fig.3A Interferon 2 CD GSE16879 IFG	unpaired Student's *t*-test	Normally distributed	Control VS Crohn's Disease colitis	t=1.618, df=23	0.1194
Fig.3A Interferon 2 CD GSE52746 IFG	unpaired Student's *t*-test	Normally distributed	Control VS Crohn's Disease colitis	t=3.277, df=25	0.0031
Fig.3A Interferon 2 CD GSE59071 IFG	unpaired Student's *t*-test	Normally distributed	Control VS Crohn's Disease colitis	t=2.299, df=17	0.0344
Fig.3A Interferon 2 CD GSE75214 IFG	unpaired Student's *t*-test	Normally distributed	Control VS Crohn's Disease colitis	t=2.275, df=17	0.0361
Fig.3A Interferon 2 UC GSE16879 IFNGR1	unpaired Student's *t*-test	Normally distributed	Control VS Ulcerative Colitis	t=0.8516, df=28	0.4017
Fig.3A Interferon 2 UC GSE37283 IFNGR1	unpaired Student's *t*-test	Normally distributed	Control VS Crohn's Disease colitis	t=3.409, df=18	0.0031
Fig.3A Interferon 2 UC GSE59017 IFNGR1	unpaired Student's *t*-test	Normally distributed	Control VS Ulcerative Colitis	t=2.083, df=106	0.0396
Fig.3A Interferon 2 UC GSE73661 IFNGR1	unpaired Student's *t*-test	Normally distributed	Control VS Crohn's Disease colitis	t=3.371, df=41	0.0016
Fig.3A Interferon 2 UC GSE75214 IFNGR1	unpaired Student's *t*-test	Normally distributed	Control VS Crohn's Disease colitis	t=4.013, df=83	0.0001

Fig.3A Interferon 2 CD GSE16879 IFNGR1	unpaired Student’s *t*-test	Normally distributed	Control VS Crohn's Disease colitis	t=0.1686, df=23	0.8676
Fig.3A Interferon 2 CD GSE52746 IFNGR1	unpaired Student’s *t*-test	Normally distributed	Control VS Crohn's Disease colitis	t=1.767, df=25	0.0895
Fig.3A Interferon 2 CD GSE59071 IFNGR1	unpaired Student’s *t*-test	Normally distributed	Control VS Crohn's Disease colitis	t=2.410, df=17	0.0275
Fig.3A Interferon 2 CD GSE75214 IFNGR1	unpaired Student’s *t*-test	Normally distributed	Control VS Crohn's Disease colitis	t=2.444, df=17	0.0257
Fig.3B STING1-UC	Brown-Forsythe and Welch ANOVA tests	Normally distributed	Normal vs. R Bef αTNF	t=4.124, df=12	4.528
Fig.3B STING1-UC	Brown-Forsythe and Welch ANOVA tests	Normally distributed	Normal vs. nR Bef αTNF	t=3.914 df=30	0.0144
Fig.3B STING1-UC	Brown-Forsythe and Welch ANOVA tests	Normally distributed	Normal vs. nR Aft αTNF	t=4.528 df=30	0.0063
Fig.3B STING1-UC	Brown-Forsythe and Welch ANOVA tests	Normally distributed	R Bef αTNF vs. R Aft αTNF	t=13 df=2.952	0.0437
Fig.3B STING1-CD	Brown-Forsythe and Welch ANOVA tests	Normally distributed	Normal vs. Bef αTNF	t=3.962 df=16	0.0144
Fig.3B STING1-CD	Brown-Forsythe and Welch ANOVA tests	Normally distributed	Normal vs. Aft αTNF	t=3.652 df=15	0.0155
Fig.3B	Brown-	Normally	Normal vs. Bef αTNF	t=3.838	0.0238

STING1-CD	Forsythe and Welch ANOVA tests	distributed		df=11	
Fig.3B STING1-CD	Brown-Forsythe and Welch ANOVA tests	Normally distributed	Normal vs. Aft αTNF	t=3.426 df=11	0.0369
Fig.3B cGAS-UC	Brown-Forsythe and Welch ANOVA tests	Normally distributed	Normal vs. nR Bef αTNF	t=4.507 df=20	0.0105
Fig.3B cGAS-UC	Brown-Forsythe and Welch ANOVA tests	Normally distributed	Normal vs. nR Aft αTNF	t=3.147 df=20	0.0429
Fig.3B cGAS-UC	Brown-Forsythe and Welch ANOVA tests	Normally distributed	nR Bef αTNF vs. nR Aft αTNF	t=2.383 df=30	0.0455
Fig.3B cGAS-CD	Brown-Forsythe and Welch ANOVA tests	Normally distributed	Normal vs. R Bef αTNF	t=3.521 df=16	0.0309
Fig.3B cGAS-CD	Brown-Forsythe and Welch ANOVA tests	Normally distributed	Normal vs. nR Bef αTNF	t=4.586 df=11	0.0109
Fig.3B cGAS-CD	Brown-Forsythe and Welch ANOVA tests	Normally distributed	R Bef αTNF vs. R Aft αTNF	t=3.134 df=21	0.0130
Fig.3B IL10-UC	Brown-Forsythe and Welch ANOVA tests	Normally distributed	Normal vs. nR Bef αTNF	t=4.339 df=20	0.0113
Fig.3B IL10-UC	Brown-Forsythe and Welch ANOVA tests	Normally distributed	Normal vs. nR Aft αTNF	t=3.912 df=20	0.0194
Fig.3B IL10-UC	Brown-Forsythe	Normally distributed	R Bef αTNF vs. nR Bef αTNF	t=2.822 df=22	0.0324

	and Welch ANOVA tests				
Fig.3B IL10-CD	Brown-Forsythe and Welch ANOVA tests	Normally distributed	Normal vs. nR Bef αTNF	t=4.158 df=11	0.0108
Fig.3B IL10-CD	Brown-Forsythe and Welch ANOVA tests	Normally distributed	Normal vs. nR Aft αTNF	t=4.611 df=11	0.0050
Fig.3B IL10-CD	Brown-Forsythe and Welch ANOVA tests	Normally distributed	R Bef αTNF vs. nR Bef αTNF	t=3.566 df=17	0.0045
Fig.3B IL1B-UC	Brown-Forsythe and Welch ANOVA tests	Normally distributed	Normal vs. R Bef αTNF	t=9.624 df=12	<0.0001
Fig.3B IL1B-UC	Brown-Forsythe and Welch ANOVA tests	Normally distributed	Normal vs. R Aft αTNF	t=4.534 df=12	0.0038
Fig.3B IL1B-UC	Brown-Forsythe and Welch ANOVA tests	Normally distributed	Normal vs. nR Bef αTNF	t=13.84 df=20	<0.0001
Fig.3B IL1B-UC	Brown-Forsythe and Welch ANOVA tests	Normally distributed	Normal vs. nR Aft αTNF	t=9.310 df=20	<0.0001
Fig.3B IL1B-UC	Brown-Forsythe and Welch ANOVA tests	Normally distributed	R Bef αTNF vs. R Aft αTNF	t=3.057 df=14	0.0160
Fig.3B IL1B-UC	Brown-Forsythe and Welch ANOVA tests	Normally distributed	R Bef αTNF vs. nR Bef αTNF	t=3.309 df=22	0.0068
Fig.3B IL1B-UC	Brown-Forsythe and Welch	Normally distributed	nR Bef αTNF vs. nR Aft αTNF	t=2.762 df=30	0.0187

	ANOVA tests				
Fig.3B IL1B-CD	Brown-Forsythe and Welch ANOVA tests	Normally distributed	Normal vs. R Bef αTNF	t=9.654 df=16	<0.0001
Fig.3B IL1B-CD	Brown-Forsythe and Welch ANOVA tests	Normally distributed	Normal vs. R Aft αTNF	t=6.020 df=15	0.0001
Fig.3B IL1B-CD	Brown-Forsythe and Welch ANOVA tests	Normally distributed	Normal vs. nR Bef αTNF	t=16.40 df=11	<0.0001
Fig.3B IL1B-CD	Brown-Forsythe and Welch ANOVA tests	Normally distributed	Normal vs. nR Aft αTNF	t=10.38 df=11	<0.0001
Fig.3B IL1B-CD	Brown-Forsythe and Welch ANOVA tests	Normally distributed	R Bef αTNF vs. R Aft αTNF	t=3.093 df=21	0.0106
Fig.3B IL1B-CD	Brown-Forsythe and Welch ANOVA tests	Normally distributed	R Bef αTNF vs. nR Bef αTNF	t=5.543 df=17	0.0001
Fig.3B IFNAR2-UC	Brown-Forsythe and Welch ANOVA tests	Normally distributed	Normal vs. R Bef αTNF	t=3.050 df=12	0.0295
Fig.3B IFNAR2-UC	Brown-Forsythe and Welch ANOVA tests	Normally distributed	Normal vs. nR Bef αTNF	t=7.047 df=22	0.0001
Fig.3B IFNAR2-UC	Brown-Forsythe and Welch ANOVA tests	Normally distributed	Normal vs. nR Aft αTNF	t=3.986 df=22	0.0053
Fig.3B IFNAR2-UC	Brown-Forsythe and Welch ANOVA	Normally distributed	R Bef αTNF vs. Aft R αTNF	t=2.925 df=14	0.0205

	tests				
Fig.3B IFNAR2-UC	Brown-Forsythe and Welch ANOVA tests	Normally distributed	R Bef αTNF vs. nR Bef αTNF	t=3.386 df=22	0.0085
Fig.3B IFNAR2-UC	Brown-Forsythe and Welch ANOVA tests	Normally distributed	nR Bef αTNF vs. nR Aft αTNF	t=3.161 df=30	0.0071
Fig.3B IFNAR2-CD	Brown-Forsythe and Welch ANOVA tests	Normally distributed	Normal vs. R Bef αTNF	t=2.789 df=15	0.0457
Fig.3B IFNAR2-CD	Brown-Forsythe and Welch ANOVA tests	Normally distributed	Normal vs. nR Bef αTNF	t=5.853 df=11	0.0007
Fig.3B IFNAR2-CD	Brown-Forsythe and Welch ANOVA tests	Normally distributed	Bef αTNF vs. Aft αTNF	t=2.869 df=20	0.0180
Fig.3B IFNAR2-CD	Brown-Forsythe and Welch ANOVA tests	Normally distributed	Bef αTNF vs. Bef αTNF	t=2.753 df=16	0.0265
Fig.4A	two-way ANOVA	Normally distributed	3% DSS mice group vs vehicle group	$F_{(7, 112)} = 17.20$	$P < 0.0001$;
Fig.4B	unpaired Student's *t*-test	Normally distributed	3% DSS mice group vs vehicle group	t=9.096 df=15	$P < 0.0001$
Fig.4C	two-way ANOVA	Normally distributed	3% DSS mice group vs vehicle group	$F_{(7, 112)} = 13.38$	$P < 0.0001$;
Fig.4D	unpaired Student's *t*-test	Normally distributed	3% DSS mice group vs vehicle group	t=17.59 df=14	$P < 0.0001$
Fig.4E	two-way ANOVA	Normally distributed	3% DSS mice group vs vehicle group	$F_{(7, 112)} = 2.444$	$P = 0.227$;
Fig.4F	unpaired Student's *t*-test	Normally distributed	3% DSS mice group vs vehicle group	t=11.42 df=14	$P < 0.0001$
Fig.4H	unpaired Student's *t*-test	Normally distributed	3% DSS mice group vs vehicle group	t=4.262 df=14	$P = 0.0008$
Fig.4L	unpaired	Normally	3% DSS mice group	t=3.281	$P =$

	Student's *t*-test	distributed	vs vehicle group	df=6	0.0168
Fig.4J	unpaired Student's *t*-test	Normally distributed	3% DSS mice group vs vehicle group	t=7.071 df=6	*P* = 0.0004
Fig.4N	unpaired Student's *t*-test	Normally distributed	3% DSS mice group vs vehicle group	t=2.376 df=8	*P* = 0.0448
Fig.4P	unpaired Student's *t*-test	Normally distributed	For Colitis group vs Normal	t=4.086 df=6	*P* = 0.0065
Fig.5A	two-way ANOVA	Normally distributed	3% DSS mice group vs vehicle group; 3% DSS + GCV group vs 3% DSS WT mice group	$F_{(21,176)} = 9.514$	$P < 0.0001$
Fig.5B	two-way ANOVA	Normally distributed	3% DSS mice group vs vehicle group; 3% DSS + GCV group vs 3% DSS WT mice group	$F_{(3, 22)} = 15.53$	$P < 0.0001$
Fig.5C	two-way ANOVA	Normally distributed	3% DSS mice group vs vehicle group; 3% DSS + GCV group vs 3% DSS WT mice group	$F_{(21, 176)} = 23.95$	$P < 0.0001$
Fig.5D	two-way ANOVA	Normally distributed	3% DSS mice group vs vehicle group; 3% DSS + GCV group vs 3% DSS WT mice group	$F_{(3, 22)} = 195$	$P < 0.0001$
Fig.5E	two-way ANOVA	Normally distributed	3% DSS mice group vs vehicle group; 3% DSS + GCV group vs 3% DSS WT mice group	$F_{(9, 88)} = 8.306$	$P < 0.0001$
Fig.5F	two-way ANOVA	Normally distributed	3% DSS mice group vs vehicle group; 3% DSS + GCV group vs 3% DSS WT mice group	$F_{(3, 22)} = 45.14$	$P < 0.0001$
Fig.5H	two-way ANOVA	Normally distributed	3% DSS mice group vs vehicle group; 3%DSS + GCV group vs 3% DSS WT mice group	$F_{(3, 22)} = 8.680$	$P = 0.0005$
Fig.5J	two-way ANOVA	Normally distributed	3% DSS mice group vs vehicle group; 3% DSS + GCV group vs 3% DSS WT mice group	$F_{(3, 12)} = 13.15$	$P = 0.0004$

Fig.5K	unpaired Student's *t*-test	Normally distributed	cGAS: GCV mice group vs vehicle group.	t = 0.09433 df = 10	*P* = 0.9267;
Fig.5K	unpaired Student's *t*-test	Normally distributed	cGAS: 3% DSS $STING^{gt/gt}$mice group vs 3% DSS WT mice group.	t = 6.190 df = 10	*P* = 0.0001;
Fig.5K	unpaired Student's *t*-test	Normally distributed	cGAS: 3% DSS + GCV mice group vs 3% DSS mice group.	t = 3.595 df= 10	*P* = 0.0049;
Fig.5K	unpaired Student's *t*-test	Normally distributed	IL-10: GCV mice group vs vehicle group.	t = 0.1566 df = 10	*P* = 0.1566;
Fig.5K	unpaired Student's *t*-test	Normally distributed	IL-10: 3% DSS $STING^{gt/gt}$mice group vs 3% DSS WT mice group.	t =5.632 df= 10	*P* = 0.0002;
Fig.5K	unpaired Student's *t*-test	Normally distributed	IL-10: 3% DSS + GCV mice group vs 3% DSS mice group.	t = 8.830 df= 10	*P* < 0.0001;
Fig.5K	unpaired Student's *t*-test	Normally distributed	IFN-β: GCV mice group vs vehicle group	t = 1.276 df = 10	*P* = 0.2308;
Fig.5K	unpaired Student's *t*-test	Normally distributed	IFN-β: 3% DSS mice group vs vehicle group	t = 6.142 df= 10	*P* = 0.0001;
Fig.5K	unpaired Student's *t*-test	Normally distributed	IFN-β: 3% DSS + GCV mice group vs 3% DSS mice group.	t = 4.518 df= 10	*P* = 0.0011;
Fig.5K	unpaired Student's *t*-test	Normally distributed	CXCL-10: GCV mice group vs vehicle group.	t = 1.073 df = 10	*P* = 0.3087;
Fig.5K	unpaired Student's *t*-test	Normally distributed	CXCL-10: 3% DSS $STING^{gt/gt}$mice group vs 3% DSS WT mice group	t = 0.6314 df= 10	*P* = 0.5419;
Fig.5K	unpaired Student's *t*-test	Normally distributed	CXCL-10: 3% DSS + GCV mice group vs 3% DSS mice group	t = 0.8310 df= 10	*P* = 0.4254;
Fig.5K	unpaired Student's *t*-test	Normally distributed	IFN-β: 3% DSS + GCV mice group vs 3% DSS mice group.	t = 4.518 df= 10	*P* = 0.0011;
Fig.5L	unpaired Student's *t*-	Normally distributed	TNF-α: GCV mice group vs	t = 0.4880 df = 10	*P* = 0.6361;

	test		vehicle group.		
Fig.5L	unpaired Student's *t*-test	Normally distributed	TNF-α: 3% DSS mice group vs vehicle group	t = 3.592 df= 10	*P* = 0.0049;
Fig.7L	unpaired Student's *t*-test	Normally distributed	TNF-α: 3% DSS + GCV mice group vs 3% DSS mice group.	t = 3.533 df= 10	*P* = 0.0054;
Fig.5L	unpaired Student's *t*-test	Normally distributed	IL-6: GCV mice group vs vehicle group.	t = 0.3636 df = 10	*P* = 0.7237;
Fig.5L	unpaired Student's *t*-test	Normally distributed	IL-6: 3% DSS mice group vs vehicle group	t = 21.82 df= 10	*P* < 0.0001 ;
Fig.5L	unpaired Student's *t*-test	Normally distributed	IL-6: 3%DSS + GCV mice group vs 3%DSS mice group.	t = 20.51 df= 10	*P* < 0.0001 ;
Fig.5L	unpaired Student's *t*-test	Normally distributed	IL-1β: GCV mice group vs vehicle group.	t = 0.3625 df = 10	*P* < 0.7245;
Fig.5L	unpaired Student's *t*-test	Normally distributed	IL-1β: 3% DSS mice group vs 3% DSS vehicle group	t = 13.42 df= 10	*P* < 0.0001 ;
Fig.5L	unpaired Student's *t*-test	Normally distributed	IL-1β: 3%DSS + GCV mice group vs 3%DSS mice group.	t = 12.49 df= 10	*P* <0.0001 ;
Fig.6B	unpaired Student's *t*-test	Normally distributed	cGAS: 3%DSS vs. vehicle group	t = 3.984 df=6	*P* = 0.0073;
Fig.6B	unpaired Student's *t*-test	Normally distributed	cGAS: GCV plus 3% DSS vs. DSS group.	t=3.101 df=6	*P* = 0.0211;
Fig.6B	unpaired Student's *t*-test	Normally distributed	STING: 3% DSS vs. vehicle group	t = 2.906 df=4	*P* = 0.0439;
Fig.6B	unpaired Student's *t*-test	Normally distributed	STING: GCV plus 3% DSS vs. DSS group.	t = 3.172 df=4	*P* = 0.0338;
Fig.6B	unpaired Student's *t*-test	Normally distributed	p-TBK1: 3% DSS vs. vehicle group	t= 5.480 df=6	*P* =0.0015;
Fig.6B	unpaired Student's *t*-test	Normally distributed	p-TBK1: GCV plus 3%DSS vs. DSS group.	t= 3.683 df=5	*P* = 0.0143;
Fig.6B	unpaired Student's *t*-test	Normally distributed	IFN-β: 3%DSS vs. vehicle group	t= 4.431 df=6	*P* =0.0044;

Fig.6B	unpaired Student's *t*-test	Normally distributed	IFN-β: GCV plus 3% DSS vs. DSS group.	t= 5.673 df=6	*P* = 0.0013;
Fig.6B	unpaired Student's *t*-test	Normally distributed	IL-1β: 3%DSS vs. vehicle group	t= 3.0215 df=6	*P* = 0.0215;
Fig.6B	unpaired Student's *t*-test	Normally distributed	IL-1β: GCV plus 3% DSS vs. DSS group.	t = 3.984 df=4	*P* = 0.0163;
Fig.6B	unpaired Student's *t*-test	Normally distributed	TNF-α: 3% DSS vs. vehicle group	t= 3.377 df=6	*P* = 0.0149 ;
Fig.6B	unpaired Student's *t*-test	Normally distributed	TNF-α: GCV plus 3% DSS vs. DSS group.	t = 3.701 df=6	*P* = 0.0101;
Fig.6D	unpaired Student's *t*-test	Normally distributed	STING: 3% DSS vs. vehicle group	t= 8.496 df=6	*P* = 0.0001 ;
Fig.6D	unpaired Student's *t*-test	Normally distributed	STING: GCV plus 3% DSS vs. DSS group.	t = 4.736 df=6	*P* = 0.0032;
Fig.7B	unpaired Student's *t*-test	Normally distributed	STING: WT group vs $STING^{gt/gt}$ group	t=5.319 df=4	*P* = 0.0060
Fig.7B	unpaired Student's *t*-test	Normally distributed	cGAS: WT group vs $STING^{gt/gt}$ group	t=3.521 df=4	*P* = 0.0244
Fig.7D	unpaired Student's *t*-test	Normally distributed	STING: WT group vs $STING^{gt/gt}$ group	t=5.379 df=6	*P* = 0.0017
Fig.7D	unpaired Student's *t*-test	Normally distributed	cGAS: WT group vs $STING^{gt/gt}$ group	t=0.5497 df=6	*P* = 0.6024
Fig.7E	two-way ANOVA	Normally distributed	3% DSS mice group vs vehicle group; 3% DSS $STING^{gt/gt}$mice group vs 3% DSS WT mice group	$F_{(21, 160)} = 21.92$	*P* < 0.0001
Fig.7F	one-way ANOVA	Normally distributed	3% DSS mice group vs vehicle group; 3% DSS $STING^{gt/gt}$mice group vs 3% DSS WT mice group	$F_{(3, 20)} = 21.92$	*P* < 0.0001
Fig.7G	two-way ANOVA	Normally distributed	3% DSS mice group vs vehicle group; 3% DSS $STING^{gt/gt}$mice group vs 3% DSS WT mice group	$F_{(21, 160)} = 17.39$	*P* < 0.0001
Fig.7H	one-way ANOVA	Normally distributed	3% DSS mice group vs vehicle group; 3%	$F_{(3, 20)} = 21.92$	*P* < 0.0001

			DSS STING$^{gt/gt}$mice group vs 3% DSS WT mice group		
Fig.7I	two-way ANOVA	Normally distributed	3% DSS mice group vs vehicle group; 3% DSS STING$^{gt/gt}$mice group vs 3% DSS WT mice group	$F_{(9, 80)} = 8.397$	$P < 0.0001$
Fig.7J	one-way ANOVA	Normally distributed	3% DSS mice group vs vehicle group; 3% DSS STING$^{gt/gt}$mice group vs 3% DSS WT mice group	$F_{(3, 20)} = 57.26$	$P < 0.0001$
Fig.7L	one-way ANOVA	Normally distributed	3% DSS mice group vs vehicle group; 3% DSS STING$^{gt/gt}$mice group vs 3% DSS WT mice group	$F_{(3, 20)} = 31.11$	$P < 0.0001$
Fig.7N	one-way ANOVA	Normally distributed	3% DSS mice group vs vehicle group; 3% DSS STING$^{gt/gt}$mice group vs 3% DSS WT mice group	$F_{(3, 12)} = 30.56$	$P < 0.0001$
Fig.7O	unpaired Student's *t*-test	Normally distributed	cGAS: 3% DSS mice group vs vehicle group	t = 3.858 df = 10	P = 0.0032;
Fig.7O	unpaired Student's *t*-test	Normally distributed	cGAS: 3% DSS STING$^{gt/gt}$mice group vs 3% DSS WT mice group	t = 3.635 df= 10	P = 0.0046;
Fig.7O	unpaired Student's *t*-test	Normally distributed	cGAS: 3% DSS mice group vs vehicle group	t = 3.308 df=10	P = 0.0079;
Fig.7O	unpaired Student's *t*-test	Normally distributed	IL-10: 3% DSS mice group vs vehicle group.	t = 2.158 df = 10	P = 0.0563;
Fig.7O	unpaired Student's *t*-test	Normally distributed	IL-10: 3% DSS STING$^{gt/gt}$mice group vs 3% DSS WT mice group	t = 4.665 df= 10	P = 0.0009;
Fig.7O	unpaired Student's *t*-test	Normally distributed	IL-10: 3% DSS mice group vs vehicle group	t =4.558 df=10	P = 0.0010;
Fig.7O	unpaired Student's *t*-test	Normally distributed	IFN-β: 3% DSS mice group vs vehicle group	t = 0.2204 df = 10	P = 0.9828;
Fig.7O	unpaired Student's *t*-	Normally distributed	IFN-β1: 3% DSS	t = 6.168 df= 10	P = 0.0001;

	test		STING$^{gt/gt}$mice group vs 3% DSS WT mice group		
Fig.7O	unpaired Student's *t*-test	Normally distributed	IFN-β1: 3% DSS mice group vs vehicle group	t =4.743 df=10	*P* =0.0008 ;
Fig.7O	unpaired Student's *t*-test	Normally distributed	CXCL-10: 3% DSS mice group vs vehicle group	t= 1.647 df= 10	*P* =0.1306 ;
Fig.7O	unpaired Student's *t*-test	Normally distributed	CXCL-10: 3% DSS STING$^{gt/gt}$mice group vs 3% DSS WT mice group	t= 1.750 df= 10	*P* = 0.1106;
Fig.7O	unpaired Student's *t*-test	Normally distributed	CXCL-10: 3% DSS mice group vs vehicle group	t= 0.3529 df=10	*P* = 0.7315;
Fig.7O	unpaired Student's *t*-test	Normally distributed	TNF-α: 3% DSS mice group vs vehicle group	t = 1.263 df= 10	*P* = 0.2353 ;
Fig.7O	unpaired Student's *t*-test	Normally distributed	TNF-α: 3% DSS STING$^{gt/gt}$mice group vs 3% DSS WT mice group	t =7.713 df=10	*P* <0.0001 ;
Fig.7O	unpaired Student's *t*-test	Normally distributed	TNF-α: 3% DSS mice group vs vehicle group	t = 7.375 df=10	*P* <0.0001 ;
Fig.7O	unpaired Student's *t*-test	Normally distributed	IL-1β: 3% DSS mice group vs vehicle group	t = 1.038 df= 10	*P* = 0.3236 ;
Fig.7O	unpaired Student's *t*-test	Normally distributed	IL-1β: 3% DSS STING$^{gt/gt}$mice group vs 3% DSS WT mice group	t = 4.383 df= 10	*P* = 0.0014 ;
Fig.7O	unpaired Student's *t*-test	Normally distributed	IL-1β: 3%DSS mice group vs vehicle group	t = 8.331 df= 10	*P* < 0.0001 ;
Fig.8F	one-way ANOVA	Normally distributed	3% DSS mice group vs vehicle group; 3% DSS STING$^{gt/gt}$mice group vs 3% DSS WT mice group	$F_{(3, 19)}$ = 5.256	*P* = 0.0082
Fig.8G	one-way ANOVA	Normally distributed	3% DSS mice group vs vehicle group; 3% DSS STING$^{gt/gt}$mice group vs 3% DSS WT mice group	$F_{(3, 19)}$ = 5.656	*P* =0.0061
Fig.8H	one-way	Normally	3% DSS mice group	$F_{(3, 19)}$ =6.082	*P* =

	ANOVA	distributed	vs vehicle group; 3% DSS STING$^{gt/gt}$mice group vs 3% DSS WT mice group		0.0044
Fig.8I	one-way ANOVA	Normally distributed	3% DSS mice group vs vehicle group; 3% DSS STING$^{gt/gt}$mice group vs 3% DSS WT mice group	$F_{(3, 19)}$ = 5.173	*P* = 0.0088
Fig.8J	one-way ANOVA	Normally distributed	3% DSS mice group vs vehicle group; 3% DSS STING$^{gt/gt}$mice group vs 3% DSS WT mice group	$F_{(3, 19)}$ = 4.099	*P* = 0.0211
Fig.8K	one-way ANOVA	Normally distributed	3% DSS mice group vs vehicle group; 3% DSS STING$^{gt/gt}$mice group vs 3% DSS WT mice group	$F_{(3, 19)}$ = 4.474	*P* = 0.0154
Fig.9A	two-way ANOVA	Normally distributed	STING$^{gt/gt}$ + DSS mice group vs STING$^{gt/gt}$ + DSS + GCV mice group	$F_{(7, 64)}$ = 0.9370	*P* =0.4846
Fig.9B	unpaired Student's *t*-test	Normally distributed	STING$^{gt/gt}$ + DSS mice group vs STING$^{gt/gt}$ + DSS + GCV mice group	t=1.054 df=8	*P* =0.3227
Fig.9C	two-way ANOVA	Normally distributed	STING$^{gt/gt}$ + DSS mice group vs STING$^{gt/gt}$ + DSS + GCV mice group	$F_{(7,64)}$ =1.502	*P* =0.1826
Fig.9D	unpaired Student's *t*-test	Normally distributed	STING$^{gt/gt}$ + DSS mice group vs STING$^{gt/gt}$ + DSS + GCV mice group	t=1.807 df=8	*P* = 0.1084
Fig.9E	two-way ANOVA	Normally distributed	STING$^{gt/gt}$ + DSS mice group vs STING$^{gt/gt}$ + DSS + GCV mice group	$F_{(3,32)}$ = 0.7662	*P* = 0.5214
Fig.9F	unpaired Student's *t*-test	Normally distributed	STING$^{gt/gt}$ + DSS mice group vs STING$^{gt/gt}$ + DSS + GCV mice group	t=1.023 df=8	*P* = 0.3361
Fig.9H	unpaired Student's *t*-test	Normally distributed	STING$^{gt/gt}$ + DSS mice group vs STING$^{gt/gt}$ + DSS + GCV mice group	t=0.6809 df=8	*P* = 0.5152
Fig.9J	unpaired Student's *t*-test	Normally distributed	STING$^{gt/gt}$ + DSS mice group vs STING$^{gt/gt}$ + DSS + GCV mice group	t=0.6547 df=6	*P* = 0.5370

Fig.9k	unpaired Student's *t*-test	Normally distributed	cGAS: $STING^{gt/gt}$ + DSS mice group vs $STING^{gt/gt}$ + DSS + GCV mice group	t=1.007 df=10	*P* = 0.3377
Fig.9k	unpaired Student's *t*-test	Normally distributed	IL-10: $STING^{gt/gt}$ + DSS mice group vs $STING^{gt/gt}$ + DSS + GCV mice group	t= 0.6384 df= 10	*P* = 0.5376
Fig.9k	unpaired Student's *t*-test	Normally distributed	IFN-β: $STING^{gt/gt}$ + DSS mice group vs $STING^{gt/gt}$ + DSS + GCV mice group	t= 0.9128 df= 10	*P* = 0.3828
Fig.9k	unpaired Student's *t*-test	Normally distributed	CXCL-10: $STING^{gt/gt}$ + DSS mice group vs $STING^{gt/gt}$ + DSS + GCV mice group	t=1.446 df=10	*P* = 0.1789
Fig.9k	unpaired Student's *t*-test	Normally distributed	TNF-α: $STING^{gt/gt}$ + DSS mice group vs $STING^{gt/gt}$ + DSS + GCV mice group	t=0.1456 df=10	*P* = 0.8871
Fig.9k	unpaired Student's *t*-test	Normally distributed	IL-6: $STING^{gt/gt}$ + DSS mice group vs $STING^{gt/gt}$ + DSS + GCV mice group	t=0.9592 df=10	*P* = 0.3601
Fig.9k	unpaired Student's *t*-test	Normally distributed	IL-1β: $STING^{gt/gt}$ + DSS mice group vs $STING^{gt/gt}$ + DSS + GCV mice group	t=0.9133 df= 10	*P* = 0.3826

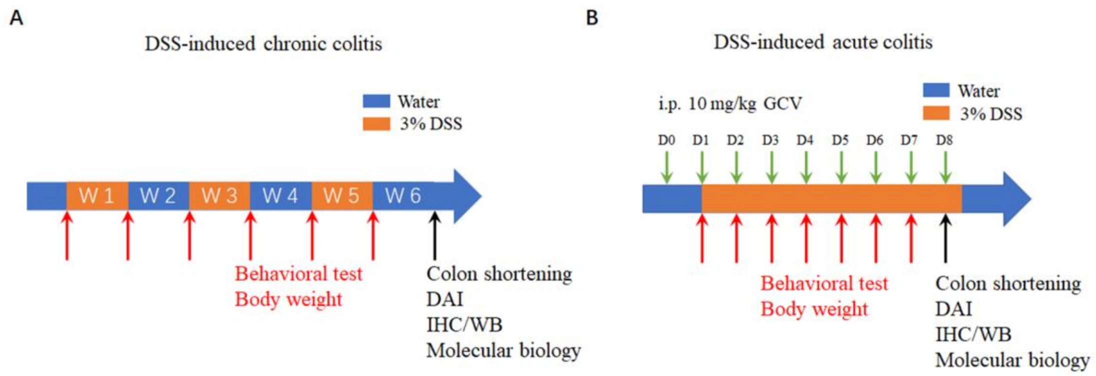

Supplementory Figure 1. The experimental precedures of DSS-induced chronic (**A**) and acute colitis (**B**) in mice.

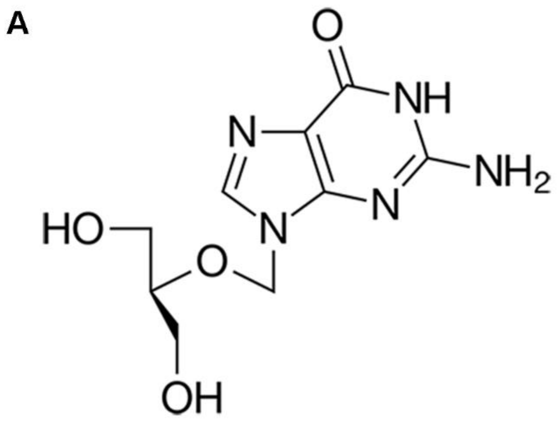

Supplementory Figure 2. (A) The molecular structure of ganciclovir.

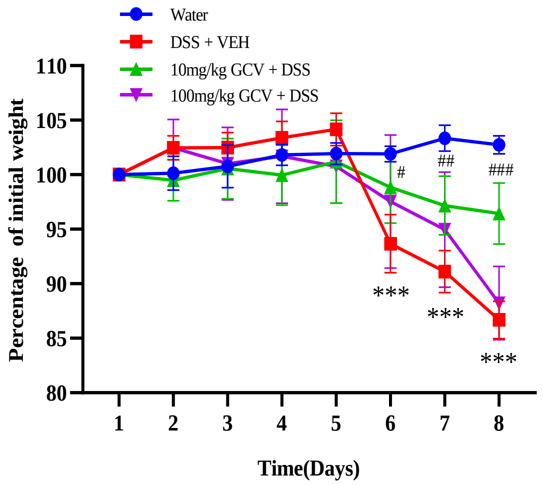

Supplementory Figure 3. The effect of different concentrations of ganciclovir on DSS-induced colitis in mice. Low-dose GCV attenuated, while high-dose GCV exacerbated DSS-colitis-induced weight loss in mice. (*n* =5-6 each group; $^{***}P < 0.001$, DSS vs. vehicle group; $^{\#}P < 0.05$, $^{\#\#}P < 0.01$, $^{\#\#\#}P < 0.001$, GCV + DSS vs. DSS group; two-way ANOVA with post-hoc Bonferroni test)

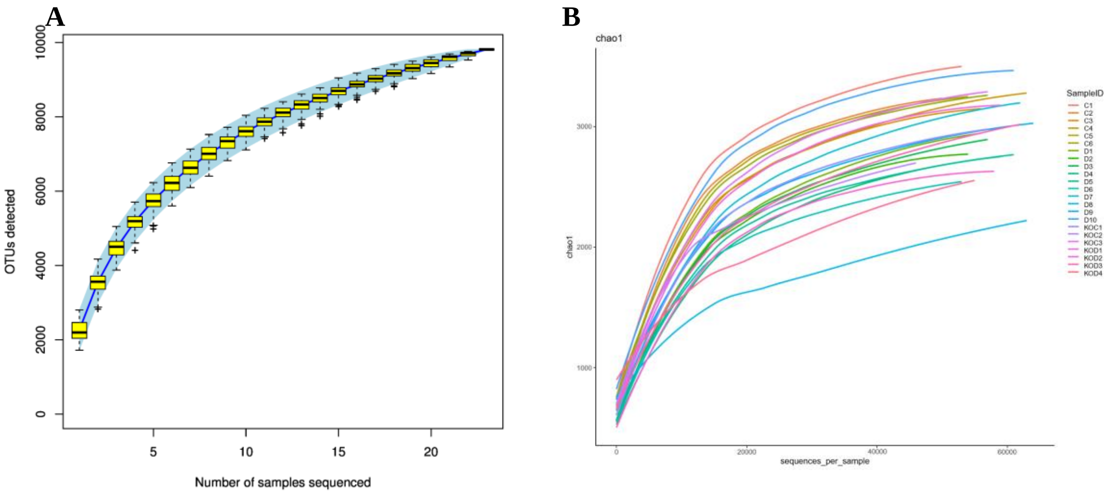

Supplementory Figure 4. Relative bacterial richness and evenness analyses. **(A)** Species accumulation curves was used to assess the number sequences likely required

to detect additional OTUs. (**B)** Rarefaction curve was used to evaluate the relative bacterial richness to determine whether further sequencing would identify additional OTUs.

A

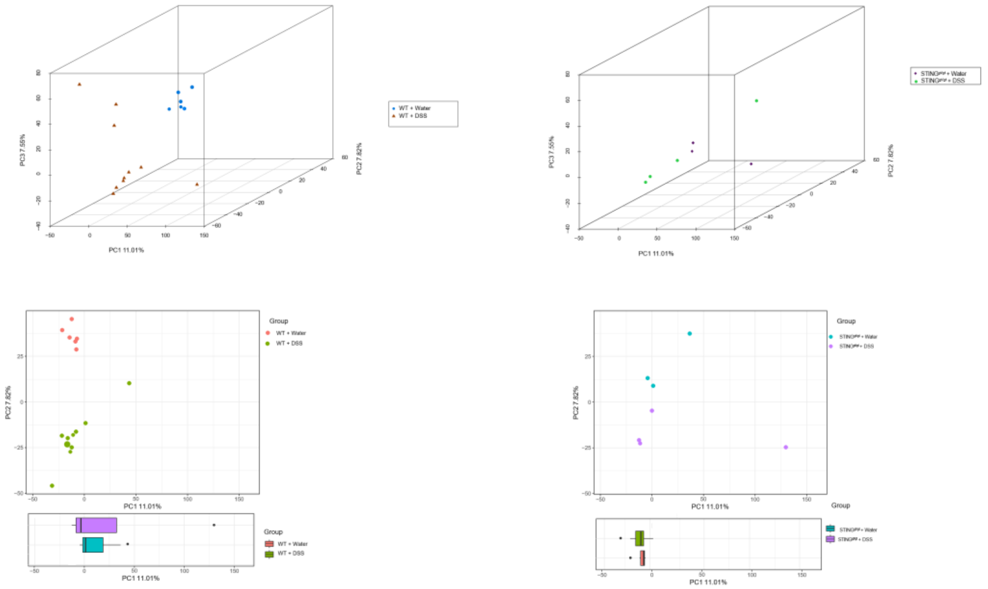

B

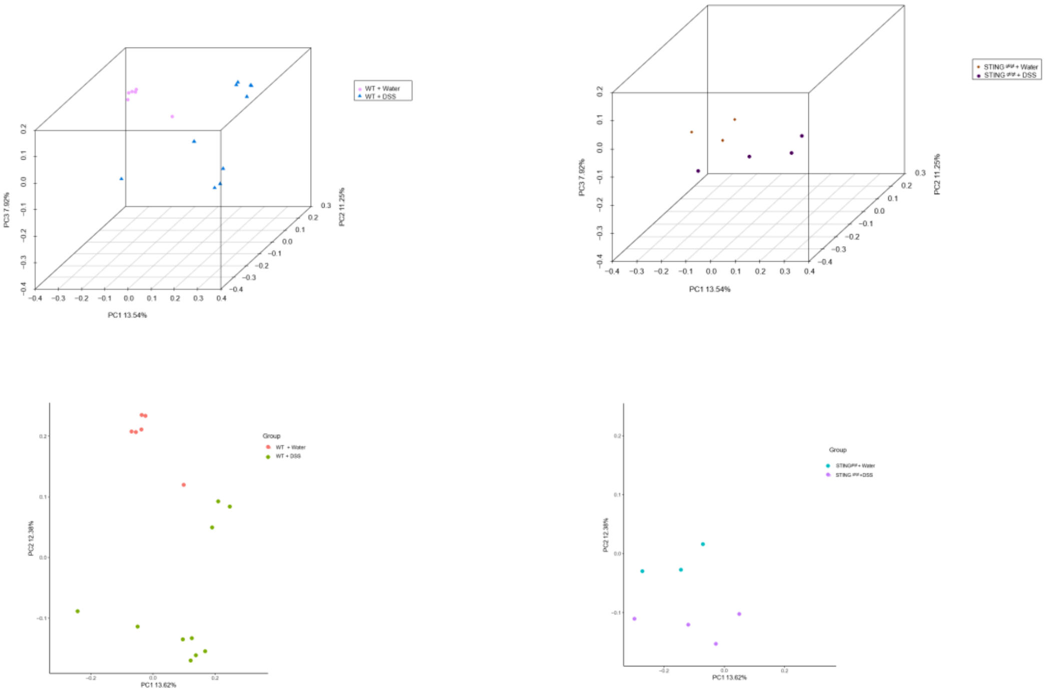

Supplementory Figure 5. (A) Assessment of structure of microbial communities by Principal Components Analysis (PCA) and **(B)** principal coordinate analyses (PCoA) plots for gut bacteria sequenced were presented.

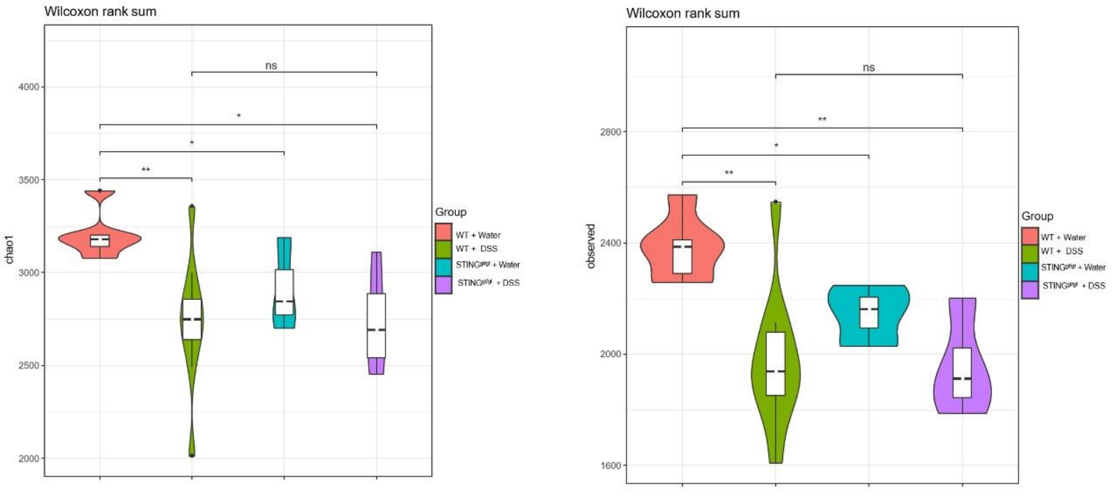

Supplementary Figure 6. The alpha diversity was assessed using the Chao1 and observed indexes, which showed significant differences among four groups. $*P < 0.05$; $^{**}P<0.01$.
